# A single sensor controls large variations in zinc quotas in a marine cyanobacterium

**DOI:** 10.1038/s41589-022-01051-1

**Published:** 2022-06-09

**Authors:** Alevtina Mikhaylina, Amira Z. Ksibe, Rachael C. Wilkinson, Darbi Smith, Eleanor Marks, James P. C. Coverdale, Vilmos Fülöp, David J. Scanlan, Claudia A. Blindauer

**Affiliations:** 1 Department of Chemistry, University of Warwick, Coventry, UK; 2 School of Life Sciences, University of Warwick, Coventry, UK; 3 Swansea University Medical School, Swansea, UK; 4 School of Pharmacy, Institute of Clinical Sciences, University of Birmingham, Birmingham, UK

**Keywords:** Metals, Proteins, Bacteria

## Abstract

Marine cyanobacteria are critical players in global nutrient cycles that crucially depend on trace metals in metalloenzymes, including zinc for CO_2_ fixation and phosphorus acquisition. How strains proliferating in the vast oligotrophic ocean gyres thrive at ultra-low zinc concentrations is currently unknown. Using *Synechococcus* sp. WH8102 as a model we show that its zinc-sensor protein Zur differs from all other known bacterial Zur proteins in overall structure and the location of its sensory zinc site. Uniquely, *Synechococcus* Zur activates metallothionein gene expression, which supports cellular zinc quotas spanning two orders of magnitude. Thus, a single zinc sensor facilitates growth across pico- to micromolar zinc concentrations with the bonus of banking this precious resource. The resultant ability to grow well at both ultra-low and excess zinc, together with overall lower zinc requirements, likely contribute to the broad ecological distribution of *Synechococcus* across the global oceans.

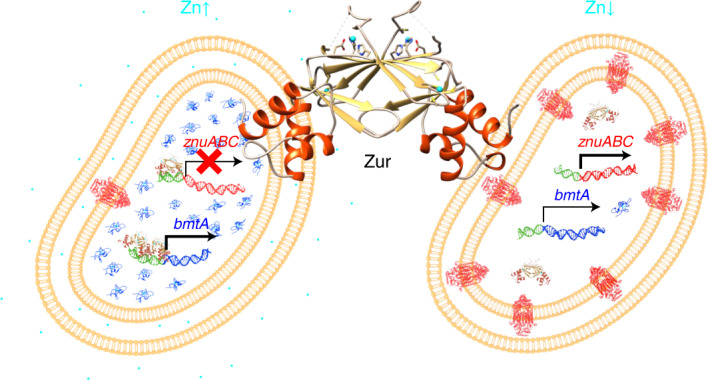

## Abstract (web summary)

The zinc-sensor protein Zur in a marine cyanobacterium is distinct from those in other bacteria in structure and location of its sensory zinc site, and facilitates growth across a range of zinc concentrations via activation of a metallothionein gene.

## Main

All major biogeochemical cycles, including those for carbon, nitrogen and phosphorus, are catalyzed by multiple enzymes, many of which require metal ions for activity^1,2^. Therefore, all organisms involved in these cycles must ensure that they acquire appropriate amounts of the entire panel of essential metals^3^. This also holds true for microorganisms that inhabit the most micronutrient-depleted regions of the open ocean, including photosynthetically active cyanobacteria of the genera *Synechococcus* and *Prochlorococcus*^4,5^. Together, these smallest but most abundant photoautotrophs contribute an estimated one-quarter of marine net primary production^6^ and hence are major drivers of the global carbon cycle^7^. Much remains to be elucidated regarding their metal ion requirements, uptake and utilization strategies.

One element that has received comparatively little attention in this context is zinc. Typically, oceanic zinc concentrations follow a nutrient-like distribution, with pico- to nanomolar concentrations in surface waters^8^. Although the importance of zinc for eukaryotic phytoplankton is undisputed^9^, evidence for the zinc limitation of open-ocean cyanobacteria is scarce^10,11^, and it has not been possible to establish whether these bacteria have an absolute requirement for zinc. On the contrary, some strains are quite sensitive to zinc toxicity, mainly due to interference with the homeostasis of other metals^12^. Nonetheless, in picoautotroph samples from the oligotrophic ocean, zinc tends to occur at similar abundance to manganese, and at levels only five to ten times lower than iron^1^, both of which are indispensable for photosynthesis. Specific metal quotas for *Synechococcus* sampled from different types of mesoscale eddies in the Sargasso Sea showed large variations in zinc quotas, ranging from 24 to 1,138 zeptomoles per cell, corresponding to tenfold lower to sevenfold higher zinc than iron quotas^13^. In addition, marine cyanobacterial genomes comprise genes encoding typically zinc-requiring enzymes such as carbonic anhydrases that are essential for effective carbon fixation, and alkaline phosphatases for phosphorus acquisition from organic substrates^14,15^. This suggests a requirement for zinc, although it is possible that these enzymes might function with other metal ions, as observed for some carbonic anhydrases from eukaryotic marine phytoplankton^16^. Further support for zinc requirement and utilization by marine cyanobacteria was provided by our previous genome-mining studies^4,17,18^, which suggested that their genomes harbor several elements of zinc homeostasis, with an emphasis on zinc uptake and storage rather than on detoxification by efflux. This conclusion also pertains to regulatory proteins, that is zinc-sensor proteins. Although homologs for the zinc excess sensor SmtB are absent from true marine strains, homologs of the ‘zinc uptake regulator’ Zur were found in every genome inspected, including a range of *Synechococcus* and *Prochlorococcus* strains, *Trichodesmium erythraeum* and *Crocosphaera watsonii*. These putative bacterial Zur proteins are members of a larger family of metal and peroxide sensors that also comprise Fur (ferric iron uptake regulator), Mur (sensing Mn^2+^), Nur (sensing Ni^2+^), Irr (sensing heme) and PerR (sensing peroxide)^19,20^. Most cyanobacteria including marine strains harbor at least three Fur-family sensors, thought to correspond to Fur, Zur and probably PerR^18^. Some biochemical and/or functional studies on the three homologs from freshwater *Anabaena* spp. PCC 7120 are available^19,21^. However, owing to the absence of structural data for any cyanobacterial Fur-family protein, no clear structure–function relationship has been established, the residues imparting metal specificity are unknown, and hence these annotations have remained tentative.

Investigations that link in vivo metal specificity with structural information for marine Fur-family sensors are therefore needed to aid our understanding of metal homeostasis and its impact on global biogeochemical cycles. Here, we provide comprehensive in vivo functional and structural data for a putative Zur protein (SYNW2401) from the model oligotrophic clade III strain *Synechococcus* sp. WH8102 (hereafter WH8102). This strain originates from the Sargasso Sea and, like other clade III *Synechococcus* strains, is well adapted to this phosphate-depleted habitat, possessing several phosphorus-related genes that are clade-specific^22,23^. Some of the encoded phosphatases may require zinc for activity^24,25^. Zinc homeostasis in this strain could, therefore, be critical to its abundance in this and other regions with low P availability. Through generation of a *zur* mutant WH8102 strain, in which the *synw2401* gene was disrupted, we establish a metal-related phenotype characterized by reduced zinc tolerance and altered zinc accumulation. Structural characterization of the recombinantly expressed SYNW2401 protein reveals unique features including a new zinc-sensing site. RNA-sequencing (RNA-seq) establishes a small set of genes regulated by Zn^2+^ and Zur, including a Zur-repressed *znuABC* uptake system and, unprecedentedly, a Zur-activated metallothionein that enables safe accumulation of intracellular Zn^2+^ and expands the range of zinc concentrations at which this strain can thrive.

## Results

### Disruption of *synw2401* alters zinc quota and tolerance

**Fig. 1:**
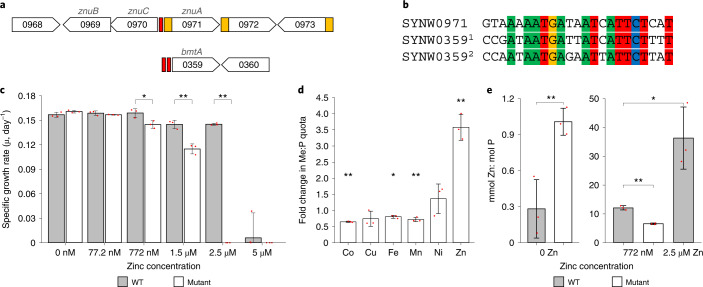
*synw2401* confers zinc tolerance and regulates zinc accumulation. **a**, Genomic locations of predicted Zur boxes (in red; retrieved from RegPrecise^26^) in WH8102. Yellow boxes denote signal sequences. The numbers refer to gene loci, that is ‘0971’ refers to *synw0971*, a predicted periplasmic metal-binding protein that forms part of an ABC-type uptake system, and ‘0359’ refers to *synw0359*, a predicted bacterial metallothionein. **b**, Sequences of the three predicted Zur boxes shown in **a**. **c**, Specific growth rates at different zinc concentrations in the medium. See Supplementary Fig. 1 for growth curves. *P* values are: 0.02497 (772 nM), 0.00305 (1.5 μM) and 2.41 × 10^−5^ (2.5 μM). **d**, Changes in metal accumulation in the mutant relative to the WT strain, determined in cells cultured at no added zinc. *P* values are: 0.00388 (Co), 0.01491 (Fe), 0.00360 (Mn) and 0.00935 (Zn). **e**, Cellular zinc quotas (expressed as mmol Zn:mol P) at three different zinc concentrations in the medium. Note the different scale for the first panel. *P* values are: 0.00935 (WT/mutant at 0 Zn), 0.00024 (WT/mutant at 772 nM Zn) and 0.01790 (WT at 772 nM/WT at 2.5 μM) See Extended Data Fig. 1c for tabulated metal quota data. In all cases, *P < 0.05 and **P < 0.01 (two-tailed *t*-test, two-sample equal variance). Data in plots **c**–**e** are presented as mean ± s.d. over *n* = 3 independent biological replicates for each condition. Source data

Previous genome-mining identified several potential players in zinc homeostasis in (marine) cyanobacteria^4,17,18^. According to our inferences, which included analysis of multiple genome neighborhoods, WH8102 harbors two putative ABC (ATP-binding-cassette)-type zinc-uptake transporter systems (ZnuABC), encoded by the genes *synw2479*–*synw2481* and *synw0969*–*synw0971*. A Zur-binding site (Zur box; Fig. 1a,b) is predicted in the intergenic region between *synw0970* (*znuC*) and *synw0971* (*znuA*)^26^, but none for *synw2479*–*synw2481*. However, two further Zur boxes are predicted in the promoter region for a gene encoding a bacterial metallothionein (*synw0359, bmtA*). Because inference of metal specificity by bioinformatics is not straightforward^18^, it is critical to experimentally establish whether the predicted Zur protein (SYNW2401) is indeed involved in zinc homeostasis and a true zinc sensor.

**Extended Data Fig. 1:**
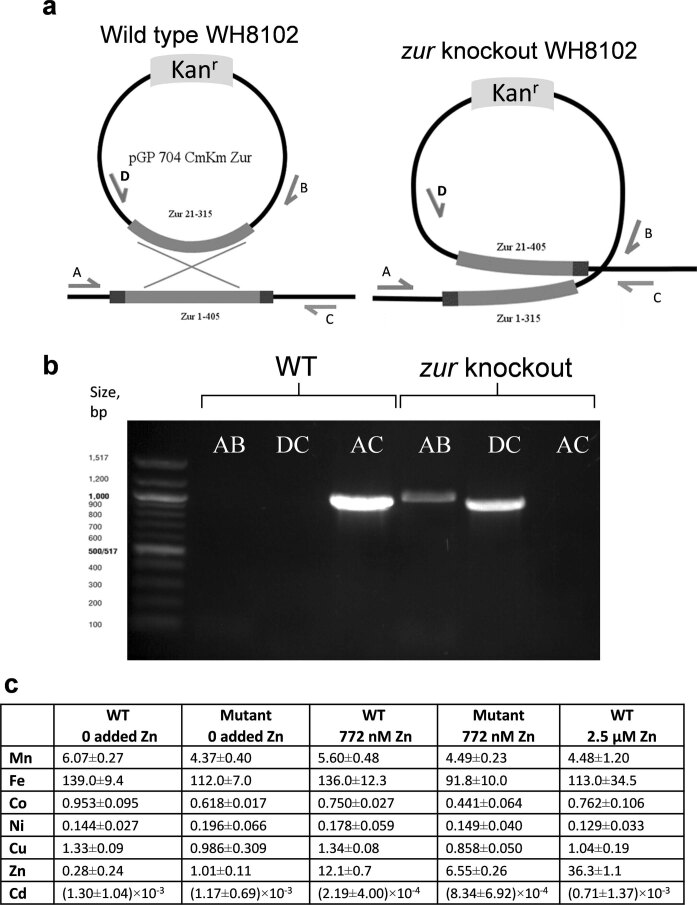
Construction, confirmation and metal accumulation of a *synw2401* disruption mutant. **a**, Construction of a single crossover *Synechococcus* sp. WH8102 *synw2401* interposon mutant. Primers used to confirm the successful mutation are indicated with letters (also see Supplementary Table 10). **b**, Agarose gel electrophoresis of PCR products obtained with these primers. This analysis was repeated multiple times (n > 3), always successfully. **c**, Cellular metal quotas, expressed as mmol metal per mol phosphorus for mutant and WT grown at different zinc concentrations. Source data

We therefore generated a single crossover interposon mutant (Extended Data Fig. 1) in which the *synw2401* gene was disrupted. The metal-specific phenotype of this mutant strain was investigated first, focusing on growth and metal accumulation when cultured in chelexed and metal-supplemented artificial seawater (ASW) medium^27^ (Fig. 1c–e). At low zinc (0 or 77.2 nM added Zn^2+^), wild-type (WT) and mutant strains grew equally well, whereas at the ‘standard’ ASW zinc concentration (772 nM^27^), the mutant began to show growth impairment (Fig. 1c and Supplementary Fig. 1). At 2.5 μM zinc, the mutant was unable to grow, in contrast to the WT which only showed relatively mild growth impairment. Further increase in [Zn] exceeded the zinc tolerance of the WT as well. Cellular metal quotas, expressed as mmol metal per mol phosphorus, were determined at two (*zur* mutant) or three (WT) zinc concentrations (Fig. 1d,e and Extended Data Fig. 1c). At both 0 and 772 nM zinc added to the culture medium, the most severely altered metal quotas were those of zinc. Notably, although media had been treated with Chelex resin, the medium with ‘0 Zn added’ evidently still supplied sufficient zinc to be accumulated in the WT and the mutant, with no indication of zinc limitation, as previously observed^28^. In all cases, the determined zinc quotas were broadly within the ranges reported for both field samples (0.5–52 mmol Zn:mol P)^13^ and laboratory cultures (0.6–8.3 mmol Zn:mol P)^29,30^ of marine *Synechococcus* and other marine cyanobacteria. Quotas for other metals were also in ranges comparable with literature data, although trending toward the high end, owing to ASW being a comparatively rich medium.

At 0 added zinc, the mutant accumulated 3.6 times more zinc than the WT, whereas the quotas of all other metals inspected either decreased (Mn, Fe, Co) or remained unchanged (Ni, Cu). These observations are consistent with SYNW2401, like other Zur proteins, repressing transcription of (at least) *znuA* (*synw0971*), encoding a periplasmic binding protein. The absence of SYNW2401 in the mutant then leads to complete de-repression of *znuA* and hence maximal zinc import through the associated ZnuABC system. The drop in the quotas of other metals may indicate the operation of compensatory processes aiming to reduce metal influx nonspecifically, or could be related to mis-metallation of sensors for other metals.

As expected, the zinc quotas of both WT and mutant increased upon addition of zinc (772 nM) to the culture medium. Zinc quotas increased by factors of 6.5 (mutant) and 43 (WT), with the WT accumulating more zinc overall than the mutant (Fig. 1e). Despite this higher cellular quota, the WT showed no growth impairment, although the mutant did. Moreover, although mild growth impairment was evident at 2.5 μM Zn, the WT was able to sustain a 129-fold increased cellular zinc quota compared with growth at 0 added Zn. This suggests that SYNW2401 also regulates a process that supports zinc accumulation without eliciting toxicity. The molecular basis for this remarkable ability to sustain zinc quotas that vary over two orders of magnitude is discussed later.

**Extended Data Fig. 2:**
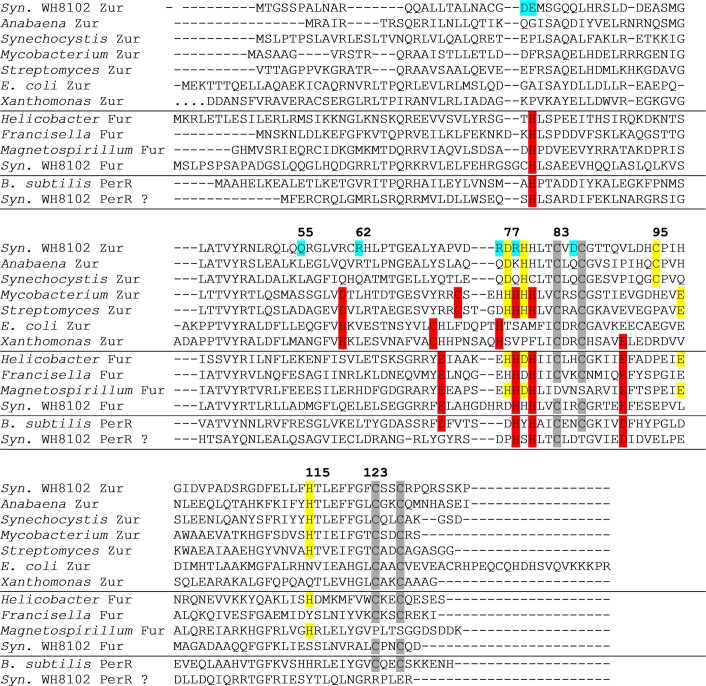
Residues critical for zinc sensing identified in other Zur proteins are not conserved in cyanobacterial homologs. Multiple sequence alignment comparing three predicted Fur-family proteins from *Synechococcus* sp. WH8102 with selected homologs for which functional and/or structural information is available. Confirmed or predicted metal-binding residues are highlighted in grey (structural ZnCys_4_ site 1), red (canonical major sensory site 2; absent in cyanobacterial Zurs), and yellow (site 3). The residues highlighted in cyan form important inter-domain or inter-subunit hydrogen bonds or salt bridges (see Fig. 3c and Extended Data Fig. 8). The other two WH8102 Fur family proteins can be predicted to possess a canonical site 2 capable of binding Fe^2+^ or Mn^2+^. Pdb entries used for metal site analysis: 2o03 (*Mycobacterium tuberculosis* FurB (Zur))^34^; 3mwm (*Streptomyces coelicolor* Zur)^39^; 4mtd (*Escherichia coli* Zur)^33^; 7dh8 (*Xanthomonas campestris* Zur; sequence lacks disordered N-terminus)^35^; 2xig (*Helicobacter pylori* Fur)^41^; 5nhk (*Francisella tularensis* Fur)^51^; 4raz (*Magnetospirillum gryphiswaldense* Fur)^40^; 3f8n (*Bacillus subtilis* PerR; Jacquamet, L. *et al*. Structural characterization of the active form of PerR: insights into the metal-induced activation of PerR and Fur proteins for DNA binding. *Mol Microbiol* **73**, 20–31 (2009).).

Thus, the WH8102 *synw2401* mutant is characterized by altered accumulation of zinc and reduced tolerance to excess zinc. Together with previous bioinformatics analyses^17,18^, the results from these phenotyping studies demonstrate that SYNW2401 indeed corresponds to the zinc sensor Zur. Because no structural information for any cyanobacterial Fur-family protein is available, and the zinc-binding residues for sensory sites known from other Zur proteins are not conserved in cyanobacterial Zurs (Extended Data Fig. 2), we determined the structure of SYNW2401 (referred to as SynZur henceforth) by X-ray crystallography.

### Cyanobacterial Zurs differ from other Zur proteins

**Fig. 2:**
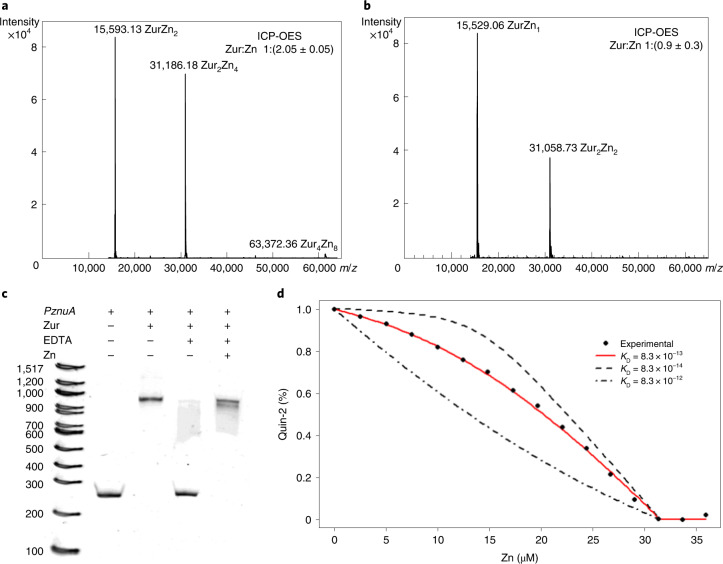
SynZur purifies with 2 Zn/monomer and binds to Zur boxes in a zinc-dependent manner. **a**, Deconvoluted native ESI-MS spectrum of SynZur as purified. The molar ratio of zinc per monomer shown in the upper right-hand corner was measured by ICP-OES. **b**, Native ESI-MS spectrum of EDTA-treated SynZur. Both ESI-MS and ICP-OES reveal the loss of one Zn/monomer. The theoretical mass of monomeric Zn_1_SynZur including six additional N-terminal residues from the cleaved tag is 15,529.79 Da (31,059.58 Da for the dimer); the measured masses are in excellent agreement with these. **c**, EMSA showing that removal of the sensory Zn^2+^ inhibits the ability of Zur to bind to *PznuA*. Reintroduction of Zn^2+^ restores the DNA-binding ability of Zur. Multiple repeats showed identical results. **d**, The sensory zinc site has an affinity for Zn^2+^ of *K*_D_ = 8.3 × 10^−13^ M, as measured by competition with Quin-2. Data points are shown as the mean ± s.e. over *n* = 3 independent technical replicates.

**Extended Data Fig. 3:**
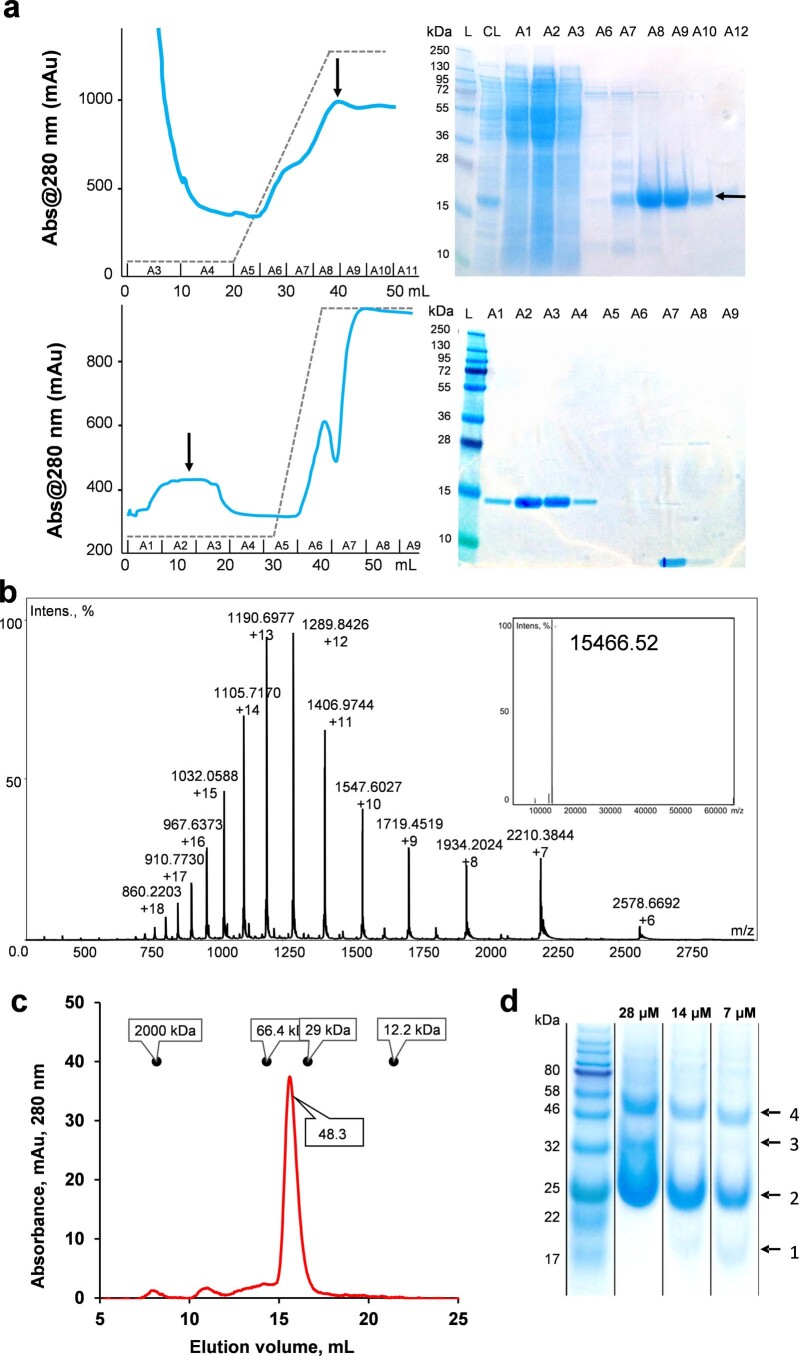
Purification and characterization of SynZur from *Synechococcus* sp. WH8102. **a**, Two-step IMAC purification of SynZur, including SDS-PAGE of fractions. Purification was repeated multiple times (n ≈ 50), always yielding the same product. **b**, The full ESI-MS spectrum of purified apo-Zur at pH 2. The deconvoluted spectrum is shown in the top right-hand corner. The pH of 5 μM SynZur in 20 mM NH_4_HCO_3_ was adjusted to 2.0 with formic acid. The theoretical neutral mass of apo-SynZur, including 6 additional residues from the cleaved tag, is 15466.43 Da. **c**, Analytical SEC of Zn_2_SynZur (40 µM) in 20 mM Tris-HCl (pH 8) and 300 mM NaCl at 0.5 mL min^−1^. Standards at concentration 1 mg mL^−1^ used were: Blue Dextran, 2000 kDa; BSA, 66.4 kDa; Carbonic anhydrase, 29 kDa; Cytochrome c, 12.2 kDa. **d**, Native SDS protein gel electrophoresis^52^ shows four bands, with the strongest band likely to correspond to a dimer. The numbers 1–4 refer to likely oligomeric states. A repeat of the experiment showed identical results. Source data

SynZur was recombinantly overexpressed with a tobacco-etch-virus-protease-cleavable His-tag in *Escherichia coli*, using standard culture medium without additional metal supplementation, and the protein was purified using an approach that avoids denaturation and metal loss (Extended Data Fig. 3). The only metal ion that was present in substantial abundance was Zn^2+^ (2 molar equivalents per subunit; Fig. 2a and Supplementary Table 1).

The molecular mass derived from size-exclusion chromatography (SEC; Extended Data Fig. 3c) did not allow conclusive derivation of the oligomeric state, but nondenaturing SDS–PAGE (Extended Data Fig. 3d) and dynamic light scattering (Supplementary Fig. 2) results are both consistent with the protein being predominantly present as a dimer. Treatment with EDTA led to the loss of one of the two bound zinc ions (Fig. 2b). This process likely corresponds to zinc sensing. Indeed, although Zn_2_SynZur as isolated binds to the *znuA* promoter (as a dimer; Supplementary Fig. 3), the presence of EDTA abolished binding (Fig. 2c). This process is reversible, because addition of Zn^2+^ to EDTA-treated SynZur re-established DNA-binding ability (Fig. 2c). The remaining zinc ion in Zn_1_SynZur likely corresponds to a ‘structural’ site; the corresponding sites in other Fur-family proteins have repeatedly been found to be refractory to removal by EDTA^20,31–35^. The Zn^2+^-binding affinity of the EDTA-responsive site, that is the sensory site, was measured by spectrophotometric titration in competition with 2-[2-[[8-[bis(carboxylatomethyl)amino]-6-methoxyquinolin-2-yl]methoxy]-*N*-(carboxylatomethyl)-4-methylanilino]acetate (Quin-2) (Fig. 2d), giving a dissociation constant (*K*_D_) of 8.3 × 10^−13^ M, similar to those measured for other Zur proteins (6.4 × 10^−13^ to 5.5 × 10^−14^ M)^20^.

**Fig. 3:**
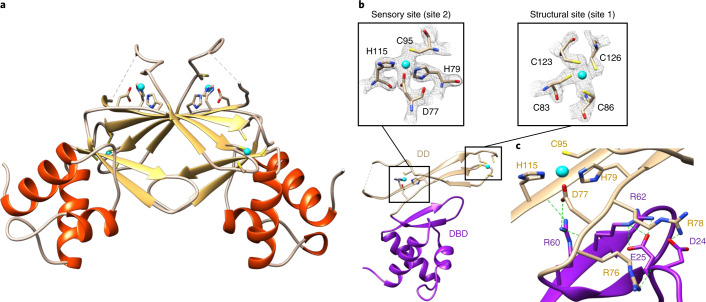
Crystal structure of SynZur from *Synechococcus* sp. WH8102. **a**, SynZur homodimer. β-strands are colored in gold and α-helices in orange. Zinc ions are shown in cyan. Residues at the N terminus up to P6, 105–107 and 129–134 are not resolved in the structure. **b**, Structural domains of the SynZur monomer and zinc-binding sites. The DBD is shown in purple and the DD in tan. The Cys_4_ site (right-hand side inset, with electron density contoured at 2.0*σ*) is conserved in many Fur-family proteins and is considered to be structural, leaving the second site (left-hand side inset, contoured at 2.0*σ*) as the sole sensory zinc site. Beyond these two sites within SynZur monomers, the crystal structure also harbors a symmetry-related zinc ion, bound to H94 and H98 of chain B in one asymmetric unit and the same two histidine residues on chain D of the adjacent unit (Extended Data Fig. 4b). The origin of this ‘surplus’ zinc ion (0.5/dimer) is unclear, but it is most likely that its presence is related to crystal packing. Inter-dimer symmetry-related zinc ions have also been observed in the structure of *Pseudomonas aeruginosa* Fur and have also been attributed a role in crystal packing^39^. Other than this zinc-bridged tetramer, no other tetrameric assemblies were suggested by PISA analysis. **c**, Hydrogen bonds and salt bridges (green) in place of the canonical zinc-sensing site 2. Residues Asp24/Glu25, Arg62 and Arg78 are in equivalent locations to three of the site 2 zinc-binding residues in other Fur-family proteins (also see Extended Data Fig. 2 for sequence alignments). It can be suggested that these electrostatic interactions stabilize the DD–DBD interface, and that Arg78 in particular communicates the presence of Zn^2+^ (cyan) in the noncanonical sensing site in SynZur to the DBD.

**Extended Data Fig. 4:**
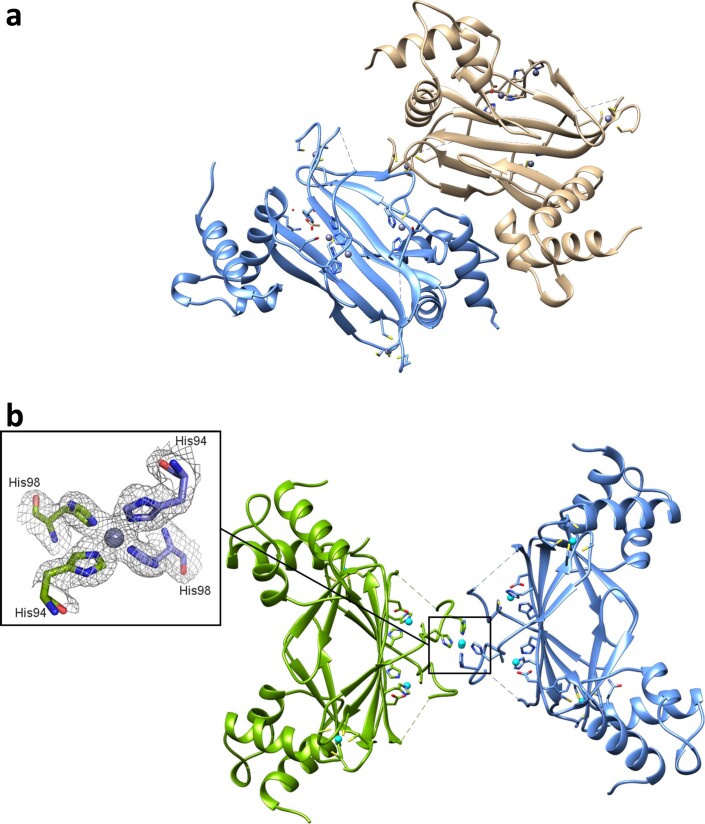
Oligomeric states in the crystal. **a**, The two dimers in the asymmetric unit of SynZur. There are no physiologically relevant contacts between these two dimers. **b**, The symmetry-related zinc site formed by chains B (green) and D (blue) of different crystallographic units. In the zoomed-in inset, the electron density map (σ level 1.5) is displayed as a mesh. The origin of this ‘surplus’ zinc ion (0.5/dimer) is unclear, but it is most likely that its presence is related to crystal packing. This assessment is based on the observation that this region is followed by a short stretch (residues 105–107 in chains A, B and D, 103–107 in chain C) with unresolved electron density. Based on structural comparisons and secondary structure predictions, residues 97–106 are expected to form an α-helix, but this is absent in the SynZur X-ray structure. We suggest that the conformational changes imposed by binding the inter-dimer zinc have caused structural disorder in this region. The non-conservation of H94 in Zur proteins from marine or freshwater cyanobacteria (Supplementary Figure 4) also argues against this zinc site relating to a physiological process, although we cannot exclude that Zur regulation in WH8102 may differ from that in other marine cyanobacteria.

Single crystals suitable for X-ray analysis were obtained in Mg(OAc)_2_/MES buffer, pH 6, from protein purified by SEC, without further addition of zinc. The structure was solved to a resolution of 2.1 Å (Supplementary Table 2) employing single-wavelength anomalous diffraction with fluorescence detected at the zinc K absorption edge (9,666 eV). This approach was necessary because molecular replacement using a range of bacterial Fur-family proteins failed—indicating that SynZur adopts a structure that substantially differs from previously determined structures. The asymmetric unit of the crystal with the space group P6_5_ contains four protein molecules. Interface analysis by PISA^36^ is consistent with SynZur forming a homodimer (Fig. 3a), with two dimers present in the asymmetric unit (Extended Data Fig. 4a). Like other Fur-family proteins^37^, each SynZur monomer consists of two domains, an N-terminal ‘winged helix’ domain that mediates interactions with DNA (DNA-binding domain (DBD); residues P6–A72) and a C-terminal domain that provides the dimerization interface (dimerization domain (DD); residues R76–P128) (Fig. 3b). The two domains are connected by a short ‘hinge’ (residues P73–D75).

**Extended Data Fig. 5:**
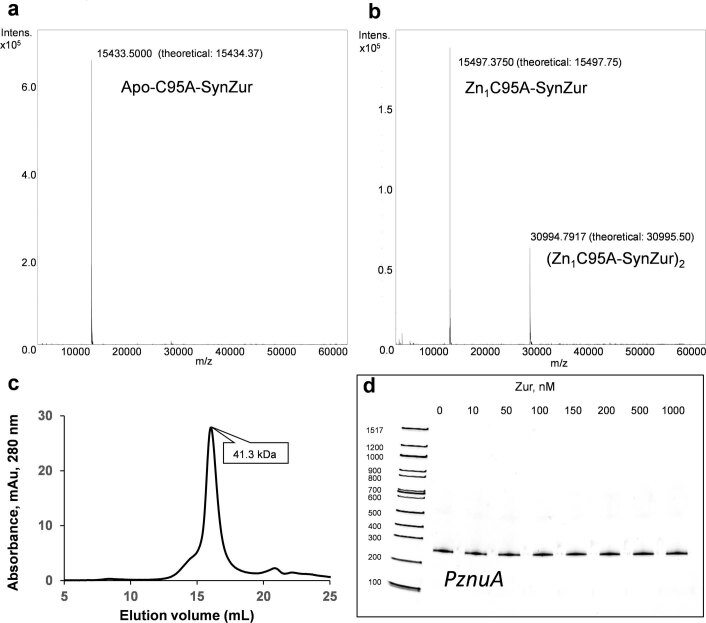
A Cys95Ala mutant loses the ability to bind to the *znuA* promoter. **a**, Deconvoluted ESI-MS spectrum under denaturing conditions (pH 2), confirming the expected mass for the C95A mutant**. b**, Deconvoluted ESI-MS spectrum under near-native conditions. The mass is consistent with that expected for the mutant protein with one zinc ion bound. Dimers are still observed in the gas phase. **c**, Analytical size-exclusion chromatogram of Cys95Ala SynZur (30 µM in 20 mM Tris-HCl (pH 8), 300 mM NaCl; Superdex G200, 10/300, GE Healthcare; 0.5 mL min^−1^). The observed mass is somewhat smaller than that for the wild-type (48.3 kDa), for which dominance of the dimer has been confirmed by dynamic light scattering analysis (Supplementary Fig. 2). We infer that the C95A mutant also still forms dimers in the solution state. **d**, The C95A mutant has no meaningful binding ability to the *znuA* promoter, similar to EDTA-treated wild-type SynZur (Fig. 2e). In contrast, wild-type SynZur as purified showed 100% binding at 100 nM. Source data

Each monomer has two zinc ions bound with bond lengths that are within the expected ranges (Supplementary Table 3); one is bound tetrahedrally by four Cys residues (83, 86, 123 and 126) and corresponds to the structural site mentioned previously (Fig. 3b; site 1). The residues forming this site are (with a single exception) 100% conserved in cyanobacterial Zur sequences (Supplementary Fig. 4). Site 1 is located in the DD and tethers the C terminus to a region close to the second zinc site, which is formed by D77, H79, C95 and H115 (site 2, Fig. 3b). To confirm that this tetrahedral site is involved in zinc sensing, we generated a Cys95Ala mutant protein (Extended Data Fig. 5). Electrospray ionization mass spectrometry (ESI-MS) analysis of this mutant showed that the purified protein retained only one zinc ion. The mutant also displayed a similar elution volume in SEC, with no indication of dissociation of the dominant dimer, indicating that loss of sensory zinc does not lead to the dissociation of the dimer at concentrations accessible to SEC. However, electrophoretic mobility shift assay (EMSA) experiments demonstrate that the mutant is unable to interact with Zur boxes (Extended Data Fig. 5d). This strongly supports the notion that site 2 is involved in zinc sensing.

**Extended Data Fig. 6:**
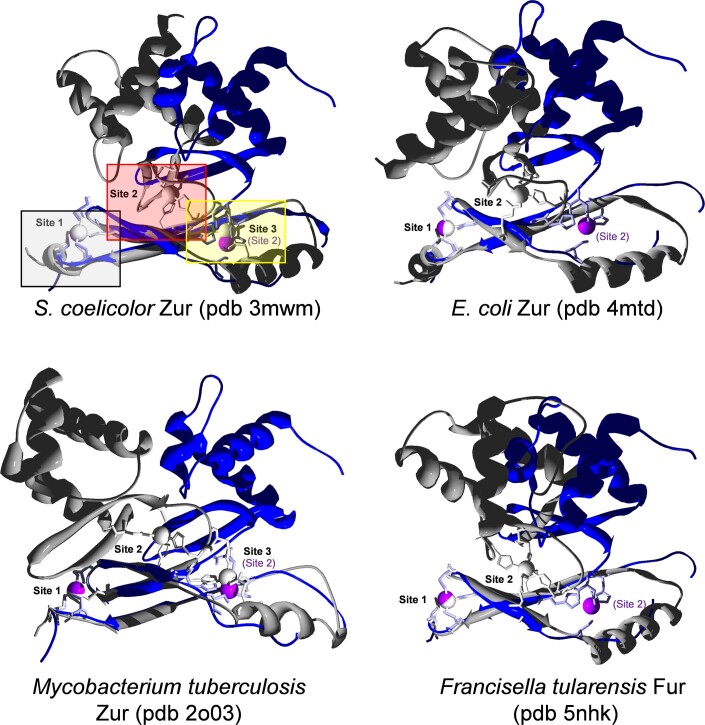
Comparison of locations of metal-binding sites on Zur and Fur proteins^33,34,39,51^. The boxes in the top left comparison are coloured to match Extended Data Fig. 2. The canonical sensing sites in other Zur and Fur proteins (grey ribbons and metal ions) is labelled ‘Site 2’, and its inter-domain location is thought to be essential to stabilise a ‘closed’ conformation with high affinity for DNA. The absence of this site in SynZur (blue ribbons and purple zinc ions) is evident. The location of the new sensory site in SynZur is similar to that of site 3 in *S. coelicolor* or *M. tuberculosis* Zur. The ligand sphere of site 3 in these proteins consists of 3 His and 1 Glu residue. In contrast, the composition of the single sensory site in SynZur (AspHis_2_Cys) resembles that of the primary zinc-sensing sites in all other characterised Zur proteins (2 His, 1 Cys and one carboxylate). All proteins shown also harbour the structural Cys_4_ site 1. For the superpositions shown, SynZur chain C (blue) was superposed with other Fur-family proteins (chain A in each case) by first matching the four Cys residues forming the structural site 1, and then improving the fits further using the respective tool in the Swiss pdb viewer v. 4.1. As can be seen, this gives a good match for the β strands in the dimerisation domain (typically around 1.2 Å RMSD for backbone heavy atoms).

The ligand sphere (N_2_OS) of this new sensory zinc site in SynZur is very similar to sites found in other Zur proteins, including the single sensing site in *E. coli*^33^ and *Xanthomonas campestris*^35^ Zur, and the primary sensing sites in *Mycobacterium tuberculosis*^38^ and *Streptomyces coelicolor* Zur^35^. However, most remarkably, the SynZur sensory site is in a location that differs from all other confirmed sensory sites in Fur-family proteins^37^ (Extended Data Figs. 2 and 6). These invariably lie between the DBD and DD^19^ involving one or two residues from the DBD, one or two from the hinge region, and one or two from the DD. This inter-domain location previously provided a straightforward understanding of the canonical sensing mechanism in Fur-family proteins: the mutual orientation between DD and DBD is not fixed in the absence of the sensory metal, whereas the presence of this metal stabilizes a conformation of the dimer in which the two DBDs are optimally oriented to match the binding sites on the cognate DNA^33,35,39–41^. By contrast, three of four of the corresponding metal-binding residues are absent in SynZur (Extended Data Fig. 2), and its sensory site does not involve any residues from the DBD (Fig. 3b). Although some Fur-family proteins harbor additional metal-binding sites in an analogous location^34–41^, SynZur is the first Fur-family protein in which this site is the sole sensory site. The absence of the canonical sensory site and the presence of this new alternative sensory site are essentially conserved in Zur proteins from both marine and freshwater cyanobacteria (Supplementary Fig. 4), with H115 being 100% conserved, H79 and C95 being fully conserved with a single exception, and D77 being present in 86.8% of sequences, occasionally (11.0%) replaced by a histidine residue, or in rare cases separated by two instead of one residue from H79. The absence of an inter-domain zinc-binding site appears to be partially compensated by a network of hydrogen bonds and salt bridges that support this conformation and may also communicate the presence of Zn^2+^ in the sensory site to the DBD (Fig. 3c).

**Extended Data Fig. 7:**
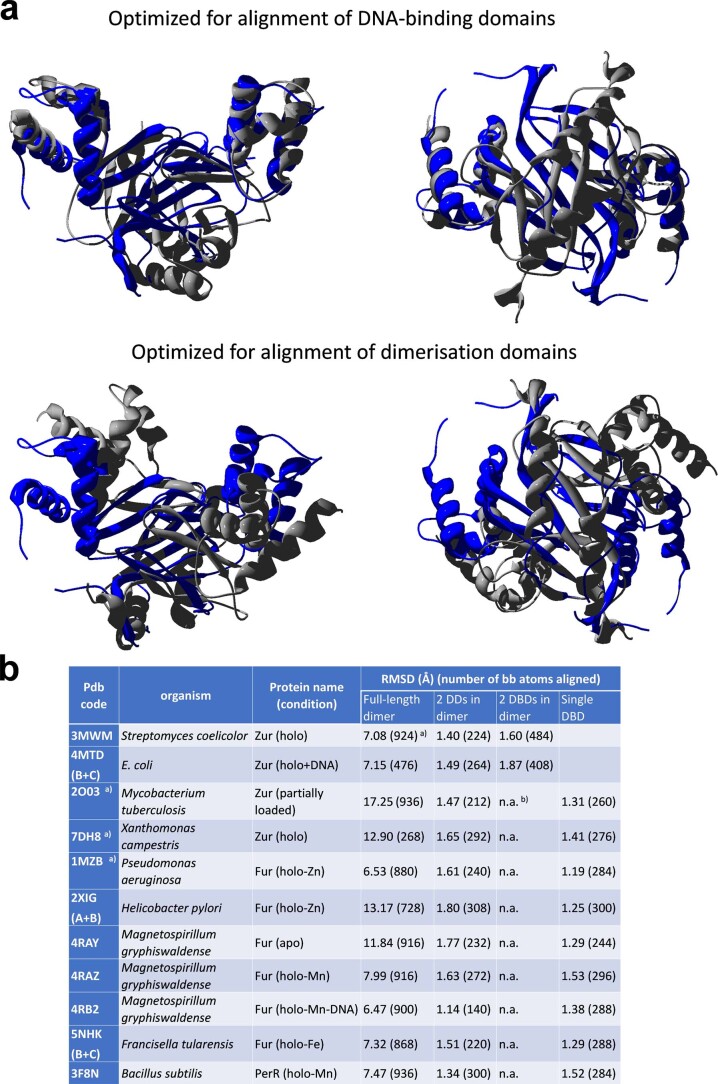
The overall structure of SynZur differs from other Fur-family proteins. **a**, Superposition of the SynZur C-D dimer (blue) with the *S. coelicolor* dimer (grey; the dimer was generated from pdb 3mwm using the PISA server). The top superpositions demonstrate that the two DBDs are in similar positions in the two dimers (121 residues across two DBDs aligned with RMSD = 1.50 Å), but that the dimerization domains are in rather different orientations. The bottom superposition shows that the dimerization domains can be aligned well (RMSD = 1.40 Å over 56 residues), but that the DBDs are in different locations relative to the dimerization domains. Superpositions were performed using the ‘explore domain alternate fit’ tool in Swiss pdb viewer v. 4.1. **b**, Structural alignment/superpositions of SynZur (dimer 2, chains C and D) with other Fur family proteins. Single domain comparisons refer to chain C for SynZur and chain A for each of the other proteins in each case. ‘Apo’ and ‘holo’ refer to occupation of sensory sites, irrespective of the presence of the ZnCys_4_ site. RMSDs for full-length dimers refer to values obtained using simple ‘magic fit’, whilst those for individual domains were obtained using the ‘explore domain alternate fit’ tool, both in Swiss pdb viewer v. 4.1. Footnotes: ^a)^ Functional dimers were generated from the original pdb files using the PISA server at https://www.ebi.ac.uk/pdbe/pisa/. Where more than two chains were present in the original pdb file, the chain identifiers are given. ^b)^ ‘n.a.’ = not applicable; this refers to cases where no superpositions were found that encompassed both DBDs.

The two DBDs in either dimer can be superimposed with the two DBDs in either *Streptomyces* Zur (1.60 Å root mean squared deviation (r.m.s.d.) over 484 backbone atoms for dimer 2 (chains C + D); Extended Data Fig. 7a) or *E. coli* Zur (1.87 Å r.m.s.d. over 408 backbone atoms; Extended Data Fig. 7b) dimers. Separately, the DDs also match well with those for other Fur-family proteins (r.m.s.d. 1.40–1.80 Å for two DDs, over 212–264 backbone atoms; Extended Data Fig. 7). However, in all cases, it is impossible to simultaneously align both DBDs and DDs in either monomer or dimer. This is due to the mutual orientation of these two domains being ‘rotated’ with respect to these other proteins (Extended Data Fig. 7a). Thus, SynZur not only harbors a new zinc-sensing site, but also displays a unique orientation of DD and DBD.

**Extended Data Fig. 8:**
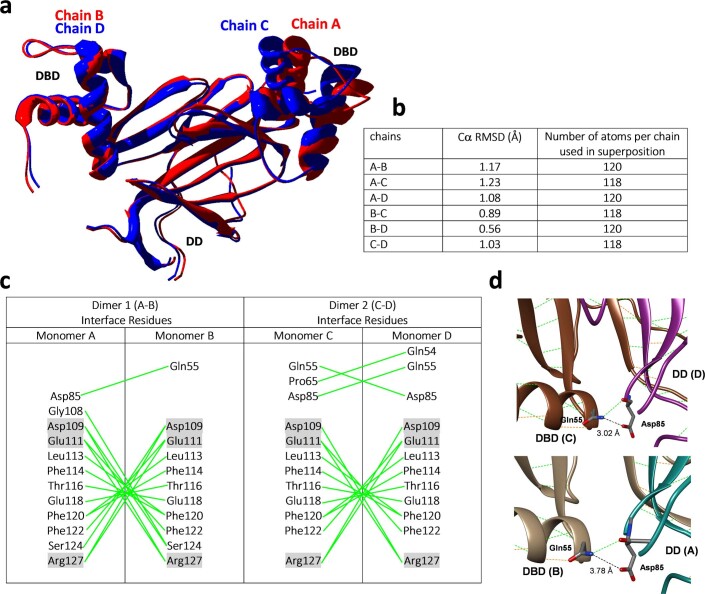
Subtle differences in structure and weak interactions between the two dimers in the crystal. **a**, Superposition of dimer 1 (chains A and B) with dimer 2 (chains C and D), where alignment of chains B and D has been optimized (carried out in Swiss pdb viewer v. 4.1). In dimer 2 (chains C and D), the two DBDs are ca. 3 Å closer together than in dimer 1 (chains A and B) This is accompanied by changes to the dimer interface and subtle variations in inter-protomer hydrogen-bonds as shown in (**b**). For dimer 1, the interface area is with 1668.3 Å^2^ slightly smaller than that for dimer 2 (1703.1 Å^2^). **b**, These differences seem to be driven by conformational dissimilarities between monomers, as comparisons between individual monomers using the SuperPose webserver (http://superpose.wishartlab.com/) shows. Chain A differs the most from other chains. (RMSDs 1.08–1.23 Å over 120 Cα carbons; **c**, Residues engaged in inter-protomer hydrogen bonds and salt bridges (green lines), as derived from analysis using PISA (https://www.ebi.ac.uk/pdbe/pisa/).^36^ The residues highlighted in grey are involved in salt bridges. **d**, Particularly noteworthy are inter-subunit H-bonds between Gln55 and Asp85. These H-bonds between the DBD of one subunit and the DD of the other may confer enhanced stability to the SynZur dimer. Again, some variability is observed in inter-subunit H-bonding between Gln55 and Asp85. For chains B, C and D, the NH_2_ of Gln55 forms an H-bond with the backbone carbonyl oxygen of Asp85, whilst for chain C, an additional interaction with the carboxylate is also likely. For chain A, Gln55 does not undergo either of these interactions.

SEC and CD spectroscopy of SynZur before and after treatment with EDTA (Supplementary Fig. 5) revealed no changes in shape, oligomerization state or secondary structure. The latter observation is not unexpected; the X-ray structures of apo- and holo-Zur from *Xanthomonas campestris* display the same secondary structure composition^35^. It is therefore likely that Zn^2+^ binding exerts more subtle effects on SynZur structural dynamics. Indeed, small differences in the conformations of the two dimers (Extended Data Fig. 8) point to a degree of conformational flexibility—even in the presence of zinc and in the crystal.

With SynZur now firmly established as a zinc sensor, we next explored its regulon in WH8102 by transcriptomic analysis.

### Zinc and SynZur regulate genes for zinc uptake and storage

**Fig. 4:**
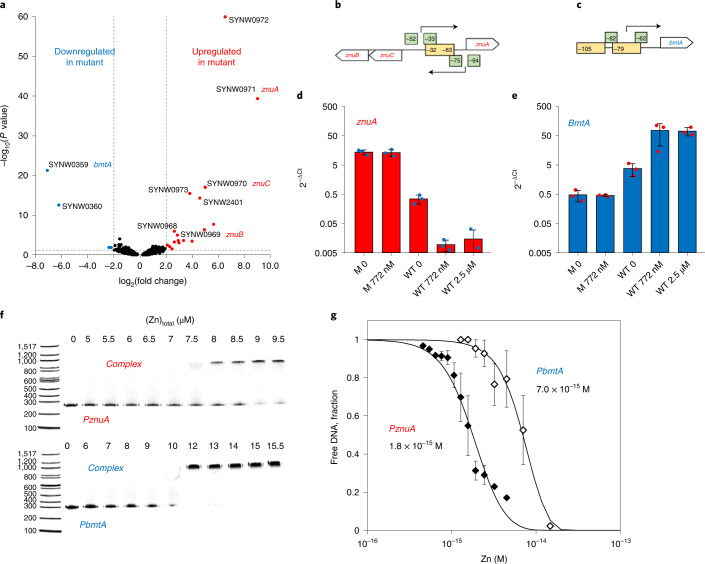
Repression and activation by SynZur. **a**, Volcano plot depicting differentially expressed genes in the mutant compared with WT at 772 nM added Zn, as determined by RNA-seq (*n* = 3 independent biological replicates). **b**,**c**, Location of −35 and −10 promoter elements (light green) relative to Zur boxes (light yellow) in the promoter regions for *znuA* and *znuC* (**b**) and *bmtA* (**c**). **d**, *ZnuA* expression (transcript abundance relative to the housekeeping gene *pepC*) in the mutant (M) and WT at different zinc concentrations quantified by RT–qPCR. For quantitative plots of *znuB* and *znuC* expression see Extended Data Fig. 10. **e**, *BmtA* expression in the mutant (M) and WT at different zinc concentrations quantified by RT–qPCR. **f**, EMSA gels for assessing the response to Zn^2+^ for SynZur binding to the *znuA* and *bmtA* promoters (5 ng promoter DNA (250 bp), 100 nM EDTA-treated SynZur, 12.5 μM *N*,*N*,*N*′,*N*′-tetrakis-(2-pyridylmethyl)ethylenediamine (TPEN). Three replicates were used to generate the data in **g**. **g**, Zn^2+^ concentration ranges for SynZur binding to *PznuA* and *PbmtA* promoters. Data in **d** and **e** are presented as mean ± s.d. over *n* = 3 independent biological replicates for each condition; each data point in **g** represents the mean ± s.e. over *n* = 3 independent replicates. Source data

**Extended Data Fig. 9:**
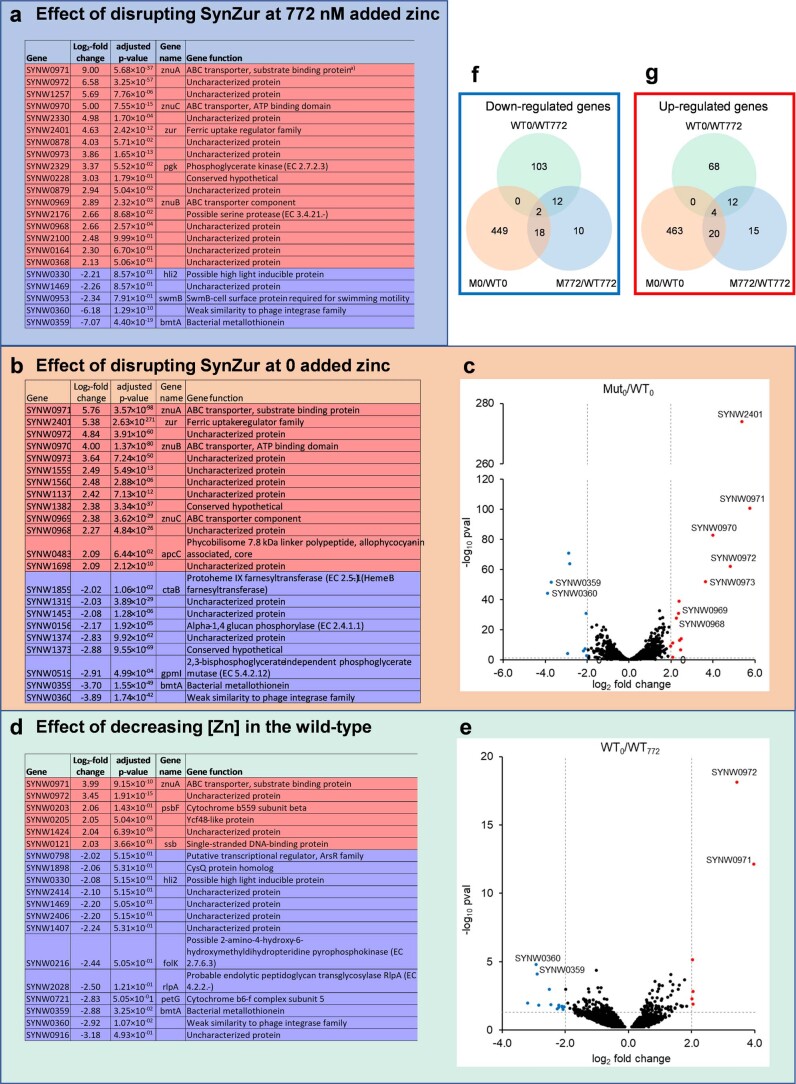
Transcriptomic analysis of mutant and wild-type *Synechococcus* sp. WH8102. Genes upregulated in the mutant (parts (**a**)-(**c**)) or at low [Zn] (parts (**d**) and (**e**)) are highlighted in red; genes downregulated in the mutant or at high [Zn] are highlighted in blue. **a**, Identity and annotations of genes that are differentially expressed in the mutant at 772 nM Zn (see Fig. 4a for Volcano plot). **b**, Identity and annotations of genes that are differentially expressed in the mutant at 0 Zn. **c**, Volcano plot depicting differentially expressed genes at 0 Zn. **d**, Identity and annotations of genes that are differentially expressed in the wild-type in dependence on [Zn]. **e**, Volcano plot depicting differentially expressed genes in the wild-type in dependence on [Zn]. In parts (**a**)-(**e**), only genes with log_2_(fold change) >2 and p-values <0.05 are highlighted and listed. **f, g**, Venn diagrams including all up- and down regulated genes where *P* < 0.05, for the three comparisons referred to in Fig. 4a and parts (**a**)-(**e**) here. We observe that *synw0969–0971* had been originally annotated with the addition ‘possibly Mn transport’. Noting that inference of metal specificity is non-trivial, we have therefore removed this incorrect specification in these Tables. Statistical analysis for parts (**a**), (**b**) and (**d**) involved default parameters for DeSeq2: Wald test for significance testing based on 3 biological replicates with two-sided *P*-values. Bonferroni cut-off was used for multiple comparisons adjustment.

**Extended Data Fig. 10:**
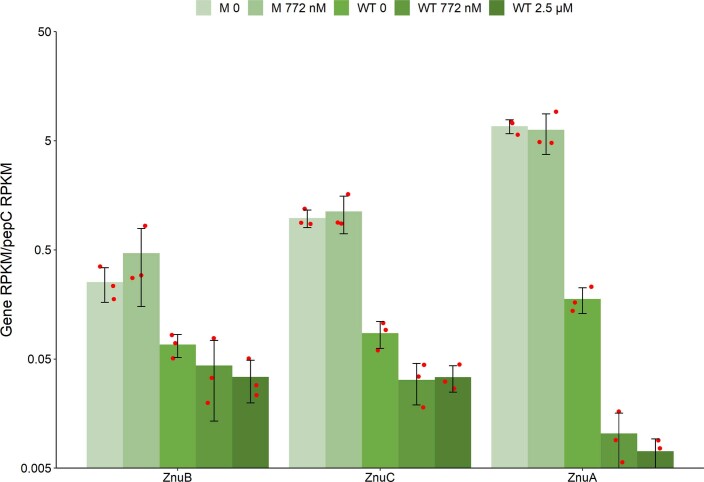
Expression levels of *znuA*, *znuB* and *znuC* from RNAseq data. The conditions (0, 772 nM, and 2.5 μM Zn^2+^ added to the culture medium) are the same as reported in Fig. 4d, e, and Extended Data Fig. 9. Transcripts (RPKM, reads per kilobase of transcript per million mapped reads) are normalized with respect to those of the *pepC* housekeeping gene. *ZnuA* and *znuBC*, being divergently transcribed, have different promoters but share a Zur box. Overall, trends are similar, but expression of *znuA* appears to be more sensitive to either Zn^2+^ or SynZur compared to those of *znuB* or *znuC*. Data are presented as mean ± standard deviation over 3 independent biological replicates for each condition.

To study SynZur-dependent transcription, mutant and WT cells were grown in chelexed ASW medium^27^, to which 0 or 772 nM Zn^2+^ had been added. Cells were harvested in mid-exponential phase (optical density at 750 nm (OD_750_) of ~0.3–0.4) and subjected to RNA-seq. Comparative data are summarized in Fig. 4 and Extended Data Figs. 9 and 10.

The most substantial changes in SynZur-dependent transcription occurred when comparing the WT and mutant at abundant zinc (772 nM; Fig. 4a and Extended Data Fig. 9a). Here, *synw0971* (putative *znuA*) was the most upregulated gene in the mutant. The fact that removal of SynZur increases expression of *znuA* is consistent with the canonical mode of action of Zur sensors, namely repression of transcription when intracellular zinc is abundant enough to bind to Zur, which in turn enhances its DNA-binding affinity^20^. In fact, the entire gene cluster *synw0968*–*synw0973*, including *znuB* (*synw0969*; encoding the permease component of the ABC transporter) and *znuC* (*synw0970*; encoding the ATPase component of the ABC transporter), was upregulated, suggesting that all six of these genes are repressed by SynZur. s*ynw0972* encodes an uncharacterized protein and is likely co-transcribed with *synw0971*. *synw0973* and *synw0968* are also annotated as uncharacterized proteins; how these are regulated by Zur is unclear. Surprisingly, *synw0359*, the bacterial metallothionein (*bmtA*), and its neighboring gene *synw0360* (‘weak similarity to phage integrase’) were both downregulated in the mutant. This suggests that these two genes are not repressed but activated by SynZur; whether this activation requires zinc-bound SynZur is explored later.

The analogous comparison at 0 nM added zinc (Extended Data Fig. 9b,c) highlighted the same eight genes, although the fold-changes were smaller in each case. The apparent upregulation of *zur* (*synw2401*) itself in the mutant is likely a consequence of the single crossover nature of the mutant constructed. RNA-seq transcripts map only to the first 315 bp of the *synw2401* gene; thus translation of this incomplete transcript would not result in a functional protein. Applying the criteria of log_2_(fold change) > 2 and *P* < 0.05, no other genes were differentially regulated at both 0 and 772 nM Zn between the WT and *zur* mutant.

Comparison of the datasets at 0 and 772 nM added Zn for the WT informs about which genes are regulated by zinc availability (Extended Data Fig. 9d,e). A relatively small number of genes were differentially regulated by more than fourfold (log_2_(fold change) > 2) between these two conditions. The most upregulated gene at 0 nM added Zn was again *synw0971*, with the adjacent *synw0972* also upregulated. The upregulation of *synw0971* in response to zinc availability further confirms that this periplasmic binding protein and its associated ABC-system components (Extended Data Fig. 10) correspond to ZnuABC, and that this system deals with zinc uptake when zinc is scarce. In turn, the two most downregulated genes at low zinc were *synw0359* (*bmtA*) and its neighbor *synw0360*, the same two genes found to be most downregulated in the *zur* mutant. This means that *bmtA* transcript levels increase at higher [Zn], suggesting that activation of transcription requires zinc-loaded SynZur. It also implies that the BmtA protein sequesters excess zinc at higher concentrations.

By contrast, *synw2401* transcript levels were not significantly altered at different zinc concentrations in the WT (*P* > 0.80), so *synw2401* transcription is not zinc-dependent, a common observation for other Zur sensors^20^. In accordance with neither Zur- nor zinc-regulation, no binding of SynZur to the *synw2401* promoter region was apparent either (Supplementary Fig. 6). This supports the suggestion that the apparent partial overexpression of *synw2401* in the mutant is a consequence of its single crossover nature.

The modulus of log_2_(fold change) for differentially expressed genes decreases in the order mutant/WT at 772 nM zinc > mutant/WT at 0 nM zinc > WT at 0/WT at 772 nM zinc. For example, for *znuA*, log_2_(fold change) values were 9.00, 5.76 and 3.99, respectively. To capture any genes that might be regulated simultaneously by zinc and SynZur, but in a less-pronounced way than specified by the log_2_(fold change) > 2 criterion, we considered all transcript level changes that fulfilled the *P* < 0.05 criterion for the three comparisons discussed so far (Extended Data Fig. 9f,g). The only two genes that were downregulated in both the absence of SynZur (irrespective of zinc supply) and at low zinc in the WT are *bmtA* and *synw0360*, and the only four upregulated genes are s*ynw0970*–*synw0973*, that is *znuC*, *znuA* and two genes encoding proteins of unknown function. *ZnuB* (*synw0969*) was upregulated 1.4-fold in the WT at 0 zinc compared with 772 nM zinc, but with very low significance (*P* = 0.83). This is also the case for the adjacent gene *synw0968*. It is likely that divergent transcription of *znuC* and *znuA* is regulated by a single Zur box (Figs. 1a and 4b). Potential RNA polymerase-binding sites identified are shown in Fig. 4b, confirming that for both *znuA* and *znuC*, the Zur box overlaps the −10 promoter elements, consistent with repression occurring through blocking RNA polymerase binding. *ZnuA* expression appears to be more sensitive than that of *znuB* or *znuC* (Extended Data Fig. 10).

The corresponding analysis for the *bmtA* promoter (Fig. 4c) indicates that the first Zur box partially overlaps both −10 and −35 elements, whereas the second box lies beyond the −35 element. Ferguson analysis of SynZur binding to the *PbmtA* promoter (Supplementary Fig. 7) indicated that at low [SynZur], only a single dimer bound, whereas at higher [SynZur], a maximum of two dimers were bound. It is unclear whether an equilibrium involving the binding of one or two dimers relates to the activation mechanism. Analysis of promoter regions of Zur-regulated genes in a range of bacteria shows that there is no discernible correlation between the presence of two *Zur* boxes and activation (Supplementary Table 4). Neither do all *bmtA* promoters from marine cyanobacteria contain two Zur boxes (Supplementary Tables 5 and 6). Our Ferguson analysis also provided no evidence for oligomerization; the latter has been observed for Zur-activated genes in *S. coelicolor*^42^ and *Xanthomonas campestris*^43^. Other possibilities for activation described for iron-responsive Fur proteins include regulation via small RNAs^38^, and via reversing H-NS silencing, as seen for ferritin expression in *E. coli*^44^. However, we were unable to find evidence for Zur/zinc regulated sRNAs or H-NS binding sites within the *PbmtA* promoter. Therefore, the mechanism of activation of *bmtA* expression by SynZur does not appear to follow any precedents. The implications of Zur-activated *bmtA* expression in response to elevated zinc availability are explored in the following section.

### Zur activation of *bmtA* enables safe accumulation of zinc

Expression patterns for *znuA* and *bmtA* were further studied by quantitative polymerase chain reaction with reverse transcription (RT–qPCR; Fig. 4d,e). These data are broadly in line with the trends observed in the RNA-seq data; maximal expression of *znuA* was observed in the mutant, irrespective of zinc concentration, followed by lower expression in the WT at 0 Zn, and very low expression at 772 nM or 2.5 μM Zn. The latter two expression levels are indistinguishable, indicating that repression is already maximal at 772 nM Zn.

The pattern for *bmtA* is essentially a mirror image of that for *znuA*, but basal expression (at low [Zn] or in the mutant) was higher than *znuA* expression at high [Zn]. At 772 nM Zn, *bmtA* transcripts were 125 times more abundant in the WT compared with the mutant (Fig. 4e). Even at 0 added Zn, the WT expressed seven times more *bmtA* than the mutant. In the WT, transcript levels at elevated Zn (772 nM or 2.5 μM) were higher by a factor of 16–17 compared with no added Zn. These data confirm that although some basal expression occurs in the mutant, Zur is required to activate *bmtA* transcription in the presence of zinc.

EMSA experiments in dependence of Zn^2+^ availability confirm that for both *znuA* and *bmtA*, Zn^2+^ is required for DNA-binding (Fig. 4f,g). The two promoters respond at slightly different free Zn^2+^ concentrations. This means that the downregulation of *znuABC* occurs at lower [Zn]_free_ than the upregulation of *bmtA*. Similar observations have been made for other Zur proteins^20,38^. It can also be suggested that the narrow range defined by the two *K*_D_ values (1.8–7.0 femtomolar) corresponds to the optimal intracellular [Zn] for *Synechococcus* sp. WH8102.

**Fig. 5:**
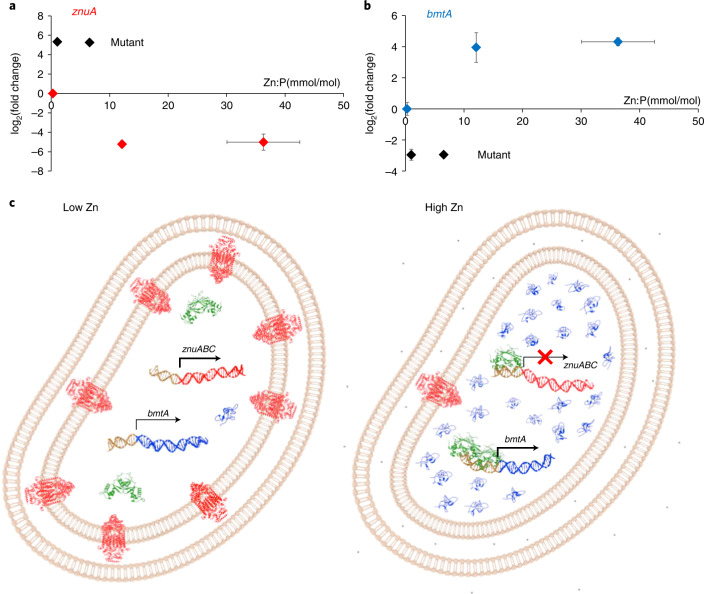
Regulation of zinc uptake and storage in WH8102. **a**, qRT–PCR data for *znuA* in dependence of accumulated zinc. All data are normalized to WT at 0 nM Zn added to the extracellular medium. In the WT, *znuA* expression is inversely correlated with accumulated Zn, whereas in the mutant, expression is high and independent of [Zn]. **b**, qRT–PCR data for *bmtA* in dependence of accumulated zinc. All data are normalized to WT at 0 nM Zn added to the extracellular medium. High expression of *bmtA* correlates with high levels of accumulated zinc. Expression (mean ± s.e.) and accumulation (mean ± s.d.) data in **a** and **b** are from *n* = 3 independent biological replicates. **c**, Overview of zinc homeostasis in WH8102. At low zinc (left), SynZur (green) does not interact with either the *znuABC* or the *bmtA* promoter. Expression of *synw0969*–*synw071* (*znuABC;* red) leads to enhanced zinc uptake. Only basal levels of BmtA (blue) are present. At high zinc (right), zinc-bound SynZur binds to both promoters, repressing *znuABC* and activating *bmtA*. Our zinc accumulation data for the WT suggest that zinc uptake still takes place when *synw0969*–*synw0971* are maximally repressed at adequate or excess zinc levels, via residual expression, nonspecific transport of zinc through other metal uptake systems or a putative second ZnuABC system^17,18^. The accumulated zinc is stored safely in overexpressed BmtA. No orthologs for zinc efflux pumps have been found in genome-mining efforts^18^. Thus, the dual regulation of uptake and storage expands the range of zinc availabilities at which *Synechococcus* sp. WH8102, and by inference other marine clade III *Synechococcus* strains, can thrive: expression of a high-affinity ZnuABC system allows adaptation to ultra-low zinc availability, whereas activation of *bmtA* enables survival at higher concentrations, perhaps with the added bonus that this allows ‘banking’ this precious resource.

Crucially, *bmtA* upregulation at higher [Zn] offers an obvious explanation (Fig. 5) as to why the WT was able to accumulate much more zinc than the mutant at 772 nM Zn while suffering no growth impairment: it can be expected that each additional BmtA protein molecule will be able to sequester up to four zinc ions^45^. Overall, this keeps the concentration of intracellular free Zn^2+^ in a safe range and allows for a 43-fold increase (Figs. 1e and 5) in the total cellular zinc quota between the 0 and 772 nM added zinc conditions in the WT.

Although these data indirectly confirm that BmtA in WH8102 has a role in dealing with zinc ‘luxury’ and excess, the observation of appreciable basal transcription of *bmtA* in both the mutant and the WT at 0 zinc (Fig. 4e) may indicate a more fundamental role for the BmtA protein, which may include redox buffering or zinc donation to other proteins^46^. Indeed, previous proteomic work investigating the response of WH8102 to phosphorus and zinc scarcity showed that the abundance of BmtA followed similar trends to those of a putative alkaline phosphatase (SYNW2391), leading to the suggestion that BmtA might supply zinc to this enzyme^25^. An analysis of the distribution of *bmtA* genes in cyanobacteria (Supplementary Table 5) may lend support to this hypothesis: *bmtA* genes are widespread in marine *Synechococcus* strains, with the majority of strains from clade III containing *bmtA* genes with two Zur boxes. The latter strains are dominant in warm oligotrophic waters^4^ that are permanently depleted in phosphorus.

## Discussion

SynZur (SYNW2401) is a metallosensor of the Fur family that responds to zinc, and hence a confirmed Zur protein. This is evidenced by: (1) impaired zinc tolerance and altered zinc accumulation in the *zur* mutant, (2) strong overlap between genes regulated by zinc and SYNW2401, (3) zinc-dependent DNA binding of the recombinantly expressed SynZur protein and (4) the presence of a sensory metal-binding site with a tetrahedral N_2_OS coordination sphere that is typical for Zn^2+^. The SynZur crystal structure is distinct from previously characterized homologs in terms of domain orientation and location of the sensory zinc site. Given the high degree of conservation between SynZur and its predicted orthologs from both marine and freshwater cyanobacteria^18^, this structure may also support further studies on any cyanobacterial Zur protein.

The Zur regulon of the marine cyanobacterium *Synechococcus* sp. WH8102 is small, comprising eight genes, six of which are repressed and two of which are activated by SynZur. Among the repressed genes are the three components of a *znuABC* Zn^2+^ uptake system (*synw0969*–*synw0971*). In contrast to repression of *znuABC*, transcriptional activation by Zur proteins is rare^20^, and Zur regulation of a bacterial metallothionein is unprecedented, as previously only transcriptional repression by SmtB-type zinc-sensor proteins has been reported^47,48^. Thus, Zur in WH8102 regulates both zinc uptake (via *znuABC*) and storage (via *bmtA*) (Fig. 5).

Taken together, our data provide evidence for zinc being an essential element for a marine cyanobacterium. The low zinc quota for the WT at 0 added zinc, together with no evidence for the cultures being zinc-limited, suggests that the minimal zinc requirements of *Synechococcus* sp. WH8102 are very low, as may be expected for an oligotrophic strain. Yet, by expressing a bacterial metallothionein, WH8102 can deploy a considerable capacity for storage of surplus zinc—up to more than two orders of magnitude above these minimal levels (Fig. 5a). Similar ranges (24 to 1,138 zeptomoles per cell) have been found for zinc quotas in marine *Synechococcus* sampled from different types of mesoscale eddies in the Sargasso Sea^13^, the original habitat of *Synechococcus* sp. WH8102. No other metal showed such a wide range. Indeed, such variations in cellular metal quotas are far from common: for example, metal quotas in *E. coli* cultured in different media including minimal^49^ and excess (0.1 mM)^50^ Zn^2+^ vary only two- to fourfold with respect to replete media.

A second putative *znuABC* system in this strain (*synw2479*–*synw2481*) was neither zinc- nor Zur-regulated. However, the periplasmic binding protein SYNW2481 was previously identified in the proteome of WH8102 cultured at 80 nM Zn^28^, and our transcriptomic data indicate that all three components are expressed at appreciable levels in all conditions. This suggests that this system is constitutively expressed and could contribute to zinc uptake even when *synw0969*–*synw0971* is completely repressed. The remarkable zinc accumulation at higher [Zn] may be facilitated either by this system and/or nonspecific transport through other metal transporters. It is also noteworthy that *synw2479*–*synw2481* are upregulated under phosphorus depletion^24^. Together with the finding of zinc-dependent abundance of an alkaline phosphatase at low [P]^25^ and the widespread distribution of Zur-regulated *bmtA* genes in clade III strains, this lends further support to the idea that zinc may be utilized for phosphorus acquisition from dissolved organic phosphates. Scavenging phosphorus from organic phosphates is a critical strategy for WH8102 and related strains being able to thrive in oligotrophic waters that are extremely scarce in phosphorus. Thus, the ability to avidly accumulate zinc when it becomes available may expand the ability of WH8102 and other oligotrophic strains that harbor *bmtA* genes to proliferate in these ‘ocean deserts’.

## Methods

### Bacterial strains and growth conditions

*Escherichia coli* cells were grown in liquid LB medium or on solid LB agar at 37 °C with kanamycin (Km, 50 μg ml^−1^), ampicillin (Amp, 100 μg ml^−1^) or chloramphenicol (Cm, 30 μg ml^−1^) added where appropriate. Strains used are shown in Supplementary Table 7.

*Synechococcus* sp. WH8102 cells were cultured in 100 ml of ASW medium without added Zn (ASW_−Zn_; Supplementary Table 8)^27^ in 250-ml glass conical flasks. Cultures were maintained at 23 °C with continuous illumination (10 μE m^−2^ s^−1^ white light) and subcultured once a month by tenfold dilution into fresh ASW_−Zn_ medium accompanied by checking for contamination. The *zur* mutant was maintained with 50 μg ml^−1^ Km.

### Construction of a single crossover *Synechococcus* sp. WH8102 *zur* mutant

Genomic DNA extracted using a phenol–chloroform protocol^51^ was used as a PCR template. Vectors used in this study are shown in Supplementary Table 9. The z*ur*_21–315_ insert was amplified using *Zur_F* and *Zur_Re* primers (Supplementary Table 10) and MyTaq Red DNA Polymerase kit (Bioline). The insert was ligated into PGP704CmKm vector at SalI and XbaI cloning sites and the mixture was transformed into *E. coli* strain S17-1 λPir. Conjugation was performed as described previously^52^ in the presence of sucrose-intolerant *Ruegeria pomeroyi* DSS-3 (pBBR-MCSI Km r pKNG101)^53^. When colonies appeared, they were transferred into 1 ml of ASW_−Zn_ containing 25 μg ml^−1^ Km and upon growth were gradually transferred into larger volumes of the medium with increasing concentrations of Km reaching 50 μg ml^−1^. Successful single crossover was assessed by colony PCR with primers *A_F* and *B_Re* or *C_Re* and *D_F* (Supplementary Table 10). Complete segregation in the mutant was assessed using PCR with primers *A_F* and *C_Re*. Completely segregated mutant cultures were incubated overnight in ASW_−Zn_ with 50 μg ml^−1^ Km, 100 μg ml^−1^ Amp and 10% (w/v) sucrose to remove *R. pomeroyi*. The mixture was then pour-plated using serial dilution to 0.22% (w/v) agarose ASW_−Zn_ with Km (50 μg ml^−1^). Single colonies were picked and again gradually transferred into larger volumes of ASW medium with Km as described above.

### Growth rate comparison

Before adding the trace metal stock (Supplementary Table 8), the ASW macronutrients solution was treated with Chelex 100 resin (Bio-Rad). *Synechococcus* sp. WH8102 WT and *zur* knockout mutant cultures were grown in chelexed ASW_−Zn_ in triplicate until the late-log phase (OD_750_ > 1). OD_750_ measurements were taken every 48–72 h until cultures reached the stationary phase. Growth experiments were repeated with different added zinc concentrations. Cultures were checked for contamination at each time point. Specific growth rates were derived from the gradients of the linear portion of growth curves. Data are the mean of three biological replicates.

### Trace metal analysis

The *zur* mutant and WT cells were grown in triplicate in chelexed ASW_−Zn_ with 0, 772 nM and 2.5 μM of zinc added. For the *zur* knockout mutant, only the 0 and 772 nM zinc conditions were used. At the mid-log stage of growth (OD_750_ = 0.4–0.5), 50 ml of cells were harvested by centrifugation at 4,000 r.p.m. for 30 min. Cells were then resuspended in 10 ml of ASW_−Zn_ with 1 mM EDTA, transferred into 15-ml Falcon tubes and centrifuged for a further 15 min. The last step was repeated twice. Finally, cell pellets were gently washed with 10 ml of MilliQ water and centrifuged, this step was repeated and the cell pellets were then snap-frozen in liquid nitrogen. These frozen cell pellets were used for inductively coupled plasma mass spectrometry (ICP-MS), RNA-seq and RT–qPCR analysis.

Subsequently, frozen cell pellets were lyophilized overnight at −65 °C until a stable weight was achieved, then digested in 300 μl of 72% ultrapure HNO_3_ overnight at 65 °C. Digests were diluted with 5.7 ml of MilliQ water to prepare samples for ICP-MS measurements of Mn, Co, Ni, Cu, Zn and Cd. For P and Fe measurements, the samples were diluted tenfold.

Standards for ICP-MS were prepared from 1,000 ppm standards (ThermoFisher Scientific) by gravimetrical dilution in 3.6% HNO_3_. The ICP-MS measurements were performed using an Agilent 7900 ICP-MS instrument in He gas mode (^31^P, ^55^Mn, ^59^Co, ^60^Ni, ^63^Cu, ^66^Zn, ^111^Cd) and H_2_ collision gas mode (^56^Fe only) with typical integration time of 1.0 s. Data were acquired and processed using Mass Hunter v.4.3 for Windows.

### RNA-seq and RT–qPCR

Frozen cell pellets obtained as described above were thawed on ice and total RNA was extracted using a phenol–chloroform protocol^54^. DNA was removed using the TURBO DNA-free kit (Ambion) and the samples were additionally purified using RNA Clean & Concentrator-5 (Zymo Research). RNA concentration and the purity of the samples were assessed using NanoDrop (ThermoFisher Scientific). The presence of DNA contamination was assessed by PCR with *16S_27F* and *16S_1492Re* rRNA gene primers (Supplementary Table 10). The RNA integrity of the samples was assessed using an Agilent Bioanalyzer with an Agilent RNA 6000 Pico Kit.

For RNA-seq analysis, RNA samples were sent to the Centre for Genomic Research, Institute of Integrative Biology at the University of Liverpool for library preparation and sequencing. RNA samples were further purified using a Qiagen RNeasy Kit. Subsequently, samples were depleted for rRNAs using a RiboZero kit (Illumina) and then dual-indexed, strand-specific RNA-seq libraries were prepared using a Next Ultra Directional RNA library preparation kit (New England Biolabs). Libraries were sequenced using an Illumina HiSeq 4000 (paired-end, 2 × 150 bp). Raw data files were trimmed for the presence of Illumina adapter sequences using Cutadapt v.1.2.1 (ref. ^55^).

For RNA-seq analysis, HISAT2 (ref. ^56^) software was used to map FASTQ reads onto the genome. Resulting SAM files were converted to BAM and sorted BAM using Samtools^57^. FeatureCounts^58^ software was used to identify mapped genes. DESeq2 (ref. ^59^) as an R-package in R-studio software was used to normalize raw reads and calculate statistics.

For RT–qPCR analysis, reverse transcription was performed using the GoScript Reverse Transcription System (Promega). The RT–qPCR mixtures were prepared in 96-well MicroAmp microplates (Applied Biosystems) and covered with MicroAmp adhesive film (Applied Biosystems). PowerUp SYBR Green Master Mix (Applied Biosystems) was used to quantify amplification. All reactions had three technical replicates for each of three biological replicates. RT–qPCR was run on a 7500 Fast Real-Time PCR System (Applied Biosystems). The presence of a single product was inspected by analysis of melting curves. Data were analyzed using 7500 software, v.2.3 (Applied Biosystems) and Microsoft Excel.

Primers for qPCR were designed using PrimerQuest Tool 150 from IDT^60^ and are given in Supplementary Table 10. The housekeeping gene *pepC* (*synw2047*, phosphoenolpyruvate carboxylase) was used to normalize transcript abundance^61^.

### SynZur overexpression and purification

The sequence for *Synechococcus* sp. WH8102 Zur was codon-optimized for expression in *E. coli* and synthesized by GeneArt (Invitrogen) before cloning into a pET155-D-TOPO vector with an N-terminal His-tag (Invitrogen). SynZur was expressed in *E. coli* BL21(DE3)pLysS (Invitrogen) grown in LB medium at 23 °C overnight following induction at the mid-log phase with 0.5 mM IPTG (ThermoFisher Scientific). Cells were lysed by sonication in Buffer I (50 mM NaH_2_PO_4_, 300 mM NaCl, 20 mM imidazole, pH 8.0). SynZur was purified using a Ni–Sepharose His-Trap column (GE Healthcare, 5 ml) using an ÄKTA purification system (GE Healthcare) with gradient elution with Buffer II (50 mM NaH_2_PO_4_, 300 mM NaCl, 250 mM imidazole, pH 8)^62^. The His-tag was cleaved by tobacco-etch-virus protease following buffer exchange into cleavage buffer (50 mM Tris–HCl pH 8.0, 1 mM DTT). Cleaved Zur was purified using the same His-Trap column. The purity of the protein was checked by SDS–PAGE in 14% Tris–glycine gel (Novex).

### Protein characterization

Analytical SEC was carried out using an ÄKTA purifier equipped with a Superdex 200, 10/300 column (GE Healthcare) running at a flow rate of 0.5 ml min^−1^ (50 mM Tris pH 8.0, 100 mM NaCl). The column was calibrated with Blue Dextran (2,000 kDa; to determine the void volume), BSA (66.4 kDa), carbonic anhydrase (29.2 kDa) and cytochrome *c* (12.2 kDa).

Dynamic light scattering was used to determine the hydrodynamic diameter of the protein. The protein was diluted to 20 µM with 50 mM Tris and filtered using a 0.2-μm pore-size filter (Sartorius Minisart RC4 syringe filter). The hydrodynamic diameter was measured at 25 °C using a Malvern Zetasizer Nano which was equilibrated for 300 s before each measurement. A total of six measurements were taken for each sample. Theoretical hydrodynamic diameters for monomers, dimers and different tetrameric assemblies were calculated from the three-dimensional structure determined in this work, with the size of an ‘intertwined’ tetramer based on the published structure for *Francisella tularensis* Fur (pdb 5nhk)^63^. For these calculations, radii of gyration (*R*_G_) were calculated using WinHydroPro^64^ and converted to hydrodynamic radii (*R*_H_) by employing the simple relationship *R*_H_ = *R*_G_/0.774 (ref. ^65^). Correct calibration of the instrument and the validity of the approach to estimate the hydrodynamic sizes were checked using carbonic anhydrase (29.2 kDa) and cytochrome *c* (12.2 kDa) measured under the same conditions.

Nondenaturing SDS–PAGE was carried out as described in ref. ^66^. Protein samples were mixed with 4× sample buffer (100 mM Tris–HCl, 150 mM Tris base, 10% v/v glycerol, 0.0185% w/v Coomassie G-250, 0.00625% w/v Phenol Red, pH 8.5). Samples were loaded onto precast 10% Tris–glycine gels, and electrophoresis was performed in nondenaturing SDS buffer (50 mM Tris pH 7.3, 50 mM MOPS, 0.0375% w/v SDS) at 4 °C and 100 V until the dye front reached the bottom of the gel. Gels were visualized using SimplyBlue SafeStain (Life Technologies) and scanned.

### Spectrophotometric determination of zinc affinity

Zinc affinity was determined following a well-established methodology suitable for metal sensors and is based on competition between apo-protein and the metallochromic dye Quin-2 (ref. ^67^). For removal of the sensory site Zn^2+^, SynZur at a concentration of 32 μM in 20 mM ammonium bicarbonate (pH 7.9) was mixed with 1 mM EDTA, 1 mM DTT and left overnight at 4 °C. The demetallated protein was purified using a PD-10 column (GE Healthcare), with two desalting runs employing 20 mM ammonium bicarbonate, pH 7.9, and all steps were carried out under an inert atmosphere. Generation of Zn_1_SynZur was ascertained by ESI-MS. Approximately 10 μM Zn_1_SynZur in 20 mM ammonium bicarbonate, pH 7.9, in the presence of 0.1 mM tris(2-carboxyethyl)phosphine (TCEP) was mixed with ~15 μM Quin-2 and titrated with 710 μM ZnSO_4_ in triplicate. The accurate Quin-2 concentration was measured spectrophotometrically at 261 nm using an extinction coefficient of 37,500 cm^−1^ M^−1^ (ref. ^68^). Protein concentration was estimated by absorbance at 280 nm, using an extinction coefficient of 3,485 cm^−1^ M^−1^. The latter was determined by accurately measuring protein concentration through sulfur quantitation by inductively coupled plasma optical emission spectroscopy (ICP-OES), and is close to the theoretical value (3,400 cm^−1^ M^−1^). The zinc concentration in stock solutions and final samples was also determined by ICP-OES. A UV–visual spectrum was measured after each addition of ZnSO_4_ repeatedly, until absorbance remained constant (up to 15 min per addition of Zn^2+^ aliquot). The *K*_D_ was calculated using DynaFit software^69^ based on Quin-2 *K*_D(Zn)_ = 3.7 × 10^−12^ M^68^.

### Generation and characterization of a Cys95Ala mutant

Mutant SynZur was generated by site-directed mutagenesis using an NEB Q5 kit and primers TCTGGATCATgcgCCGATTCATGGTATTGATGTTCCGG (forward; the lower-case “gcg” shows the mutated codon for Ala) and ACCTGGGTGGTGCCACAA (reverse). Expression and purification of the Cys95Ala protein followed the same protocols as for the WT, with protein mass determined by ESI-MS.

### DNA-binding experiments

For EMSAs a 252-bp fragment from the intergenic region between *synw0970* (*znuC*) and *synw0971* (*znuA*), was amplified by PCR, purified and diluted in EMSA binding buffer (10 mM Tris, pH 8, 50 mM KCl, 2 mM MgCl_2_·6H_2_O, 5% glycerol, 0.05 mg ml^−1^ BSA, 1 mM DTT, 3 mM spermidine). Primers used were *pznuABC_F* and *pznuABC_Re* (Supplementary Table 10). Aliquots containing 5 ng of DNA were mixed with various concentrations of SynZur or Cys95Ala SynZur and made up to 10 μl with EMSA binding buffer followed by incubation at room temperature for 30 min. Reaction mixtures were loaded onto precast 10% polyacrylamide gels (0.1 M Tris, pH 8.3) and then PAGE was performed in Tris–glycine buffer (0.025 and 0.187 M, respectively) at 100 V for ~90 min at 4 °C.

DNA was visualized with SYBR Green (Sigma-Aldrich) diluted 1:10,000 in Tris–glycine buffer using a luminescent image analyzer (ImageQuant LAS 4000; GE Healthcare Bio-Sciences AB).

The stoichiometry of protein–DNA complexes was assessed using Ferguson plots, adopting methodology from ref. ^70^. Protein standards (P77125 or P7719S; New England Biolabs Color Prestained Protein Standard, Broad Range) were run together with DNA–SynZur complexes using cast gels with various acrylamide concentrations under the standard EMSA conditions described above. In addition, 5 μl of MyTaq red buffer containing an inert dye of low molecular mass was loaded into a separate well as a low mass control. After SYBR Green staining, gels were first scanned in transillumination mode to visualize the protein ladder and low molecular mass control before changing to fluorescence mode for DNA visualization. The two gel images were combined using GIMP v.2.8.10. The mobility of DNA bands in pixels was measured using the ‘Measure tool’ in GIMP. Negative slopes for mobility in dependence on gel percentage for each standard, free DNA and DNA–protein complexes were derived and plotted in dependence of molecular mass, using the standards to derive a linear fit.

To determine the response of *znuA* and *bmtA* promoters to Zn^2+^, each 20-µl reaction contained 12.5 µM TPEN (*N*,*N*,*N*ʹ,*N*ʹ-tetrakis-(2-pyridylmethyl)ethylenediamine), 100 nM Zn_1_SynZur, 5 ng of *PznuA* or *PbmtA* promoters and variable ZnSO_4_ in Tris–glycine buffer with 20% glycerol (pH 8), chelexed before use. Mixtures were equilibrated for 30 min, loaded onto pre-run Novex WedgeWell 10%–20% Tris–glycine gels and subjected to PAGE in chelexed Tris–glycine buffer at 4 °C and 100 V for 90 min. Gels were stained for 30 min in SYBR Green solution and visualized as described above. Bands were quantified using ImageJ, and data were fitted in Dynafit software.

### Crystallization, data collection and structure determination

Zur was purified as described above, with a final SEC purification step using a Sephacryl S-200 column (HiPrep 26/60, GE Healthcare) in 50 mM Tris–HCl pH 8, 150 mM NaCl. Fractions containing SynZur were pooled and concentrated (Amicon Ultra, 3 kDa molecular weight cutoff) to 10 mg ml^−1^. Screening of crystallization conditions was performed with a TPP Labtech Mosquito robot using various commercial screens in MRC 96-well plates. Initial hits were observed in well F3 of the Proplex screen (Molecular Dimensions) at 18 °C. Crystallization conditions required further optimization for well-diffracting crystals. Final crystals were grown in a hanging drop format with 1 μl of protein mixed with 1 μl of crystallization solution and incubated at 4 °C. Small rod-shaped crystals appeared after 1 week, grown in 100 mM magnesium acetate, 100 mM MES pH 6, 16% PEG 10000. Crystals were harvested using a 0.08-mm mounted Litholoop (Molecular Dimensions), cryoprotected in crystallization solution containing 20% ethylene glycol and flash-frozen in liquid nitrogen.

X-Ray diffraction data to a resolution of 2.1 Å were collected at the zinc absorption edge (9,666 eV) at beamline I03, using a Pilatus 6 M detector, at the Diamond Light Source, Didcot, UK. All data were indexed, integrated and scaled using the XDS package^71^. Further data handling was carried out using the CCP4 software package^72^. The structure was solved by single-wavelength anomalous diffraction using SHELX^73^, which identified all nine Zn^2+^ ions in the crystallographic unit cell. The resulting model was further extended and refined by alternate cycles of manual refitting using Coot^74^ and Refmac^75^. Water molecules were added to the atomic model automatically using ARP^76^, at the positions of large positive peaks in the difference electron density map, only at places where the resulting water molecule fell into an appropriate hydrogen-bonding environment. Restrained isotropic temperature factor refinements were carried out for each individual atom. The polypeptide chain was traced continuously through electron density maps (2mFo–ΔFc and mFo–ΔFc) from residues 6–104 and 108–128 for chains A, B and D, and residues 6–102 and 108–128 for chain C, respectively. Data collection and refinement statistics are given in Supplementary Table 2.

### Promoter analyses

The 150 bp promoter regions of marine cyanobacterial metallothionein genes were extracted manually from Cyanorak^77^. Putative cyanobacterial Zur-binding box was inferred from RegPrecise^25^ (NTNANAATGATNATCATTNTNAN). Scanning across cyanobacterial metallothionein promoters was performed using FIMO (part of the MEME suite) with default parameters^78^. Bacterial genes with predicted double Zur boxes were extracted from RegPrecise. The −10, −35 elements were identified by Softberry BPROM^79^.

### Reporting summary

Further information on research design is available in the Nature Research Reporting Summary linked to this article.

## Online content

Any methods, additional references, Nature Research reporting summaries, source data, extended data, supplementary information, acknowledgements, peer review information; details of author contributions and competing interests; and statements of data and code availability are available at 10.1038/s41589-022-01051-1.

## Supplementary information

Supplementary Information Supplementary Tables 1–10 and Figs. 1–7.

Reporting Summary

## Data Availability

*E. coli* strains (Supplementary Table 7), plasmids (Supplementary Table 9) and oligonucleotides (Supplementary Table 10) are provided in the Supplementary Information. The atomic coordinates and structure factors for *Synechococcus* sp. WH8102 Zur have been deposited in the Protein Data Bank under the accession number 7NE9. RNA-sequencing data have been deposited in the EMBL-EBI ArrayExpress database under the accession number E-MTAB-10194. RNA-seq FASTQ files are available at http://cgr.liv.ac.uk/illum/LIMS16056_259a058713a41d9b/. Source data are provided with this paper. Other data supporting our findings are available in extended data and supplementary information. Any raw biophysical and RT–qPCR and construct DNA sequencing data are available from the corresponding author c.blindauer@warwick.ac.uk.

## Source data

Source Data Fig. 1 Unprocessed gel.
Source Data Fig. 4 Unprocessed gels.
Source Data Extended Data Fig. 1 Unprocessed gel.
Source Data Extended Data Fig. 3 Unprocessed gels.
Source Data Extended Data Fig. 5 Unprocessed gel.

## Code availability

R-Studio scripts used for RNA-seq analysis are available at https://github.com/AlevtinaMikh/Syn8102.

## References

[1] Twining BS, Baines SB (2013). The trace metal composition of marine phytoplankton. Annu. Rev. Mar. Sci..

[2] Andreini C, Bertini I, Rosato A (2009). Metalloproteomes: a bioinformatic approach. Acc. Chem. Res..

[3] Waldron KJ, Robinson NJ (2009). How do bacterial cells ensure that metalloproteins get the correct metal?. Nat. Rev..

[4] Scanlan DJ (2009). Ecological genomics of marine picocyanobacteria. Microbiol. Mol. Biol. Rev..

[5] Biller SJ, Berube PM, Lindell D, Chisholm SW (2015). *Prochlorococcus*: the structure and function of collective diversity. Nat. Rev. Microbiol..

[6] Flombaum P (2013). Present and future global distributions of the marine cyanobacteria *Prochlorococcus* and *Synechococcus*. Proc. Natl Acad. Sci. USA.

[7] Lee, C.-T. A., Jiang, H., Dasgupta, R. & Torres, M. in *Deep Carbon: Past to Present* (eds Orcutt, B. et al.) 313–357 (Cambridge Univ. Press, 2019).

[8] Middag R, de Baar HJW, Bruland KW (2019). The relationships between dissolved zinc and major nutrients phosphate and silicate along the GEOTRACES GA02 transect in the West Atlantic Ocean. Glob. Biogeochem. Cycles.

[9] Sunda WG (2012). Feedback interactions between trace metal nutrients and phytoplankton in the ocean. Front. Microbiol..

[10] Saito MA, Sigman DM, Morel FMM (2003). The bioinorganic chemistry of the ancient ocean: the co-evolution of cyanobacterial metal requirements and biogeochemical cycles at the Archean–Proterozoic boundary?. Inorg. Chim. Acta.

[11] Sunda WG, Huntsman SA (1995). Cobalt and zinc interreplacement in marine phytoplankton: biological and geochemical implications. Limnol. Oceanogr..

[12] Hawco NJ, Saito MA (2018). Competitive inhibition of cobalt uptake by zinc and manganese in a pacific *Prochlorococcus* strain: insights into metal homeostasis in a streamlined oligotrophic cyanobacterium. Limnol. Oceanogr..

[13] Twining BBS (2010). Variations in *Synechococcus* cell quotas of phosphorus, sulfur, manganese, iron, nickel, and zinc within mesoscale eddies in the Sargasso Sea. Limnol. Oceanogr..

[14] Aizawa K, Miyachi S (1986). Carbonic anhydrase and CO_2_ concentrating mechanisms in microalgae and cyanobacteria. FEMS Microbiol. Lett..

[15] Moore LR, Ostrowski M, Scanlan DJ, Feren K, Sweetsir T (2005). Ecotypic variation in phosphorus-acquisition mechanisms within marine picocyanobacteria. Aquat. Microb. Ecol..

[16] Lane TW (2005). A cadmium enzyme from a marine diatom. Nature.

[17] Blindauer CA (2008). Zinc-handling in cyanobacteria: an update. Chem. Biodivers..

[18] Barnett JP (2012). Mining genomes of marine cyanobacteria for elements of zinc homeostasis. Front. Microbiol..

[19] Fillat MF (2014). The FUR (ferric uptake regulator) superfamily: diversity and versatility of key transcriptional regulators. Arch. Biochem. Biophys..

[20] Mikhaylina A, Ksibe AZ, Scanlan DJ, Blindauer CA (2018). Bacterial zinc uptake regulator proteins and their regulons. Biochem. Soc. Trans..

[21] Sein-Echaluce VC (2018). Molecular basis for the integration of environmental signals by FurB from *Anabaena sp PCC 7120*. Biochem. J..

[22] Palenik B (2003). The genome of a motile marine *Synechococcus*. Nature.

[23] Doré H (2020). Evolutionary mechanisms of long-term genome diversification associated with niche partitioning in marine picocyanobacteria. Front. Microbiol..

[24] Ostrowski M (2010). PtrA is required for coordinate regulation of gene expression during phosphate stress in a marine *Synechococcus*. ISME J..

[25] Cox AD, Saito MA (2013). Proteomic responses of oceanic *Synechococcus* WH8102 to phosphate and zinc scarcity and cadmium additions. Front. Microbiol..

[26] Novichkov PS (2013). RegPrecise 3.0—a resource for genome-scale exploration of transcriptional regulation in bacteria. BMC Genomics.

[27] Wilson WH, Carr NG, Mann NH (1996). The effect of phosphate status on the kinetics of cyanophage infection in the oceanic cyanobacterium *Synechococcus* sp. WH7803. J. Phycol..

[28] Barnett JP, Scanlan DJ, Blindauer CA (2014). Identification of major zinc-binding proteins from a marine cyanobacterium: insight into metal uptake in oligotrophic environments. Metallomics.

[29] Quigg A (2003). Evolutionary inheritance of elemental stoichiometry in phytoplankton. Nature.

[30] Köbberich M, Vance D (2019). Zn isotope fractionation during uptake into marine phytoplankton: implications for oceanic zinc isotopes. Chem. Geol..

[31] Jacquamet L (1998). X-ray absorption spectroscopy of a new zinc site in the Fur protein from *Escherichia coli*. Biochemistry.

[32] Althaus EW, Outten CE, Olson KE, Cao H, O’Halloran TV (1999). The ferric uptake regulation (Fur) repressor is a zinc metalloprotein. Biochemistry.

[33] Gilston BA (2014). Structural and mechanistic basis of zinc regulation across the *E. coli* Zur regulon. PLoS Biol..

[34] Lucarelli D (2007). Crystal structure and function of the zinc uptake regulator FurB from *Mycobacterium tuberculosis*. J. Biol. Chem..

[35] Liu F (2021). Structural basis for zinc-induced activation of a zinc uptake transcriptional regulator. Nucleic Acids Res..

[36] Krissinel E, Henrick K (2007). Inference of macromolecular assemblies from crystalline state. J. Mol. Biol..

[37] Sarvan S, Butcher J, Stintzi A, Couture JF (2018). Variation on a theme: investigating the structural repertoires used by ferric uptake regulators to control gene expression. Biometals.

[38] Shin JH (2011). Graded expression of zinc-responsive genes through two regulatory zinc-binding sites in Zur. Proc. Natl Acad. Sci. USA.

[39] Pohl E (2003). Architecture of a protein central to iron homeostasis: crystal structure and spectroscopic analysis of the ferric uptake regulator. Mol. Microbiol..

[40] Deng Z (2015). Mechanistic insights into metal ion activation and operator recognition by the ferric uptake regulator. Nat. Commun..

[41] Dian C (2011). The structure of the *Helicobacter pylori* ferric uptake regulator Fur reveals three functional metal binding sites. Mol. Microbiol..

[42] Choi SH (2017). Zinc-dependent regulation of zinc import and export genes by Zur. Nat. Commun..

[43] Huang DL (2008). The Zur of *Xanthomonas campestris* functions as a repressor and an activator of putative zinc homeostasis genes via recognizing two distinct sequences within its target promoters. Nucleic Acids Res..

[44] Nandal A (2010). Induction of the ferritin gene (*ftnA*) of *Escherichia coli* by Fe^2+^-Fur is mediated by reversal of H-NS silencing and is RyhB independent. Mol. Microbiol..

[45] Blindauer CA (2011). Bacterial metallothioneins: past, present, and questions for the future. J. Biol. Inorg. Chem..

[46] Blindauer, C. A. in *Binding, Transport and Storage of Metal Ions in Biological Cells* (eds Maret W. & Wedd, A.) Ch. 21 (Royal Society of Chemistry, 2016).

[47] Thelwell C, Robinson NJ, Turner-Cavet JS (1998). An SmtB-like repressor from *Synechocystis* PCC 6803 regulates a zinc exporter. Proc. Natl Acad. Sci. USA.

[48] Liu T (2004). A novel cyanobacterial SmtB/ArsR family repressor regulates the expression of a CPx-ATPase and a metallothionein in response to both Cu(I)/Ag(I) and Zn(II)/Cd(II). J. Biol. Chem..

[49] Outten CE, O’Halloran TV (2001). Femtomolar sensitivity of metalloregulatory proteins controlling zinc homeostasis. Science.

[50] Xu ZL (2019). Zinc excess increases cellular demand for iron and decreases tolerance to copper in *Escherichia coli*. J. Biol. Chem..

[51] Murray MG, Thompson WF (1980). Rapid isolation of high molecular weight plant DNA. Nucleic Acids Res..

[52] Brahamsha B (1996). A genetic manipulation system for oceanic cyanobacteria of the genus *Synechococcus*. Appl. Environ. Microbiol..

[53] Aguilo-Ferretjans M (2021). Pili allow dominant marine cyanobacteria to avoid sinking and evade predation. Nat. Commun..

[54] Logemann J, Schell J, Willmitzer L (1987). Improved method for the isolation of RNA from plant tissues. Anal. Biochem..

[55] Martin M (2011). Cutadapt removes adapter sequences from high-throughput sequencing reads. EMBnet J..

[56] Kim D, Langmead B, Salzberg SL (2016). HISAT: a fast spliced aligner with low memory requirements Daehwan. Nat. Methods.

[57] Li H (2009). The Sequence Alignment/Map format and SAMtools. Bioinformatics.

[58] Liao Y, Smyth GK, Shi W (2014). FeatureCounts: an efficient general purpose program for assigning sequence reads to genomic features. Bioinformatics.

[59] Love MI, Huber W, Anders S (2014). Moderated estimation of fold change and dispersion for RNA-seq data with DESeq2. Genome Biol..

[60] PrimerQuest Tool. IDT https://eu.idtdna.com/PrimerQuest/Home/Index (2022).

[61] Eriksson, V. I. *The Response of* Synechococcus *sp. CC9311 to Iron Stress.* PhD thesis, University of Warwick (2013).

[62] Tropea, J. E., Cherry, S. & Waugh, D. S. in *High Throughput Protein Expression and Purification, Methods and Protocols* (ed. Doyle, S. A.) 297–307 (Humana Press, 2009).

[63] Pérard J (2016). Quaternary structure of Fur proteins, a new subfamily of tetrameric proteins. Biochemistry.

[64] Ortega A, Amoros D, de la Torre JG (2011). Prediction of hydrodynamic and other solution properties of rigid proteins from atomic- and residue-level models. Biophys. J..

[65] Smilgies DM, Folta-Stogniew E (2015). Molecular weight-gyration radius relation of globular proteins: a comparison of light scattering, small-angle X-ray scattering and structure-based data. J. Appl. Crystallogr..

[66] Nowakowski AB, Wobig WJ, Petering DH (2014). Native SDS-PAGE: high resolution electrophoretic separation of proteins with retention of native properties including bound metal ions. Metallomics.

[67] VanZile ML, Chen X, Giedroc DP (2002). Structural characterization of distinct α3N and α5 metal sites in the cyanobacterial zinc sensor SmtB. Biochemistry.

[68] Jefferson JR, Hunt JB, Ginsburg A (1990). Characterization of Indo-1 and Quin-2 as spectroscopic probes for Zn^2+^–protein interactions. Anal. Biochem..

[69] Kuzmič P (1996). Program DYNAFIT for the analysis of enzyme kinetic data:application to HIV proteinase. Anal. Biochem..

[70] Baichoo N, Helmann JD (2002). Recognition of DNA by Fur: a reinterpretation of the Fur box consensus sequence. J. Bacteriol..

[71] Kabsch W (2010). XDS. Acta Crystallogr. D Biol. Crystallogr..

[72] Dodson EJ, Winn M, Ralph A (1997). Collaborative computational project, number 4: providing programs for protein crystallography. Methods Enzymol..

[73] Sheldrick GM (2008). A short history of SHELX. Acta Crystallogr. A.

[74] Emsley P, Cowtan K (2004). Coot: model-building tools for molecular graphics. Acta Crystallogr. D Biol. Crystallogr..

[75] Murshudov GN, Vagin AA, Dodson EJ (1997). Refinement of macromolecular structures by the maximum-likelihood method. Acta Crystallogr. D Biol. Crystallogr..

[76] Langer G, Cohen SX, Lamzin VS, Perrakis A (2008). Automated macromolecular model building for X-ray crystallography using ARP/wARP version 7. Nat. Protoc..

[77] Garczarek L (2021). Cyanorak v2.1: a scalable information system dedicated to the visualization and expert curation of marine and brackish picocyanobacteria genomes. Nucleic Acids Res..

[78] Grant CE, Bailey TL, Noble WS (2011). FIMO: scanning for occurrences of a given motif. Bioinformatics.

[79] Solovyev, V. & Salamov, A. V. in *Metagenomics and its Applications in Agriculture* (ed. Li, R. W.) 61–78 (Nova Science, 2010).

