# Supplementary material for: A single sensor controls large variations in zinc quotas in a marine cyanobacterium
Source: Nat Chem Biol. 2022 Jun 9;18(8):869–77. doi: 10.1038/s41589-022-01051-1 (PMC9337993; doi:10.1038/s41589-022-01051-1)
Supplement: Supplementary file 1 — Supplementary Tables 1–10 and Figs. 1–7. [file 41589_2022_1051_MOESM1_ESM.pdf]

nature portfolio

https://doi.org/10.1038/s41589-022-01051-1

Supplementary information

A single sensor controls large variations in zinc quotas in a marine cyanobacterium

In the format provided by the
authors and unedited

Supplementary Information

A single sensor controls large variations in zinc quotas in a marine cyanobacterium

Alevtina Mikhaylina[1,2], Amira Z. Ksibe[1,2], Rachael C. Wilkinson[2,3], Darbi Smith[1], Eleanor Marks[1], James P. C. Coverdale[1,4], Vilmos Fülöp[2], David J. Scanlan[2], Claudia A. Blindauer[1]

[1] Department of Chemistry, University of Warwick, UK

[2] School of Life Sciences, University of Warwick, UK

[3] Swansea University Medical School, Swansea, UK

[4] School of Pharmacy, Institute of Clinical Sciences, University of Birmingham, UK

Email: c.blindauer@warwick.ac.uk

Contents

Supplementary Table 1. Metal contents of recombinantly expressed WH8102 Zur (ICP-OES).

Supplementary Table 2. X-ray crystallography data collection and refinement statistics.

Supplementary Table 3. Average metal distances in the crystal structure of WH8102 Zur at the two zinc sites.

Supplementary Table 4. Genes with promoters that harbour two or more Zur boxes in a range of bacterial genomes.

Supplementary Table 5. Distribution of *bmtA* and putative Zur-regulated *znuA* genes in *Synechococcus* strains.

Supplementary Table 6. Predicted Zur boxes in cyanobacterial metallothionein promoters.

Supplementary Table 7. *Escherichia coli* strains used in this work.

Supplementary Table 8. Composition of ASW_{-Zn} medium.

Supplementary Table 9. Plasmids used in this work.

Supplementary Table 10. PCR primers used in this study. Restriction sites are highlighted.

Supplementary Figure 1. Growth curves for wild-type and mutant *Synechococcus* sp. WH8102 at different zinc concentrations in the medium

Supplementary Figure 2. Estimation of oligomerisation state of Zn_2SynZur by dynamic light scattering.

Supplementary Figure 3. Ferguson plot analysis of SynZur binding to the *PznuA* promoter.

Supplementary Figure 4. The four residues of the new sensory site are conserved in putative cyanobacterial Zur proteins.

Supplementary Figure 5. SEC and CD data for EDTA-treated SynZur.

Supplementary Figure 6. WH8102 Zur does not bind to the *zur* (synw_2401) promoter region.

Supplementary Figure 7. Ferguson plot analysis of SynZur binding to the *PbmtA* promoter.

Supplementary Table 1. Metal content of recombinantly expressed SynZur (ICP-OES). Ratios were calculated based on protein concentration determined via simultaneously measured sulfur concentration (Data are mean ± standard deviation from n = 3 independent replicates).

**Metal, M**	**M to protein ratio**	**St. error**
**Zn**	**2.053**	**0.005**
Ni	0.017	0.005
Cd	0.003	0.003
Cu	0.008	0.001
Mn	0.010	0.002

Supplementary Table 2 | Data collection and refinement statistics

**Data collection**	
Space group	$P6_5$
Cell dimensions	
a, *b*, *c* (Å)	129.25, 129.25, 77.28
α, β, γ (°)	90, 90, 90
Wavelength (Å)	1.2827
Resolution (Å)	77.3-2.1 (2.21-2.1)*
R_{sym} or R_{merge}	0.193 (2.55)
$I / \sigma I$	19.4 (2.0)
Completeness (%)	99.9 (99.6)
Redundancy	39.8 (39.7)
$CC_{1/2}$	99.9 (75.4)
**Refinement**	
No. reflections	43002
R_{work} / R_{free}	0.194 / 0.218
No. atoms	
Protein	3712
Zn^{2+}	9
Water/Acetate	144
B-factors	
Protein	47.7
Zn^{2+}	45.2
Water/Acetate	49.2
R.m.s deviations	
Bond lengths (Å)	0.014
Bond angles (°)	1.6

Values are for a single crystal.

*Values in parentheses are for highest-resolution shell.

Supplementary Table 3. Average metal distances in the crystal structure of WH8102 Zur at the two zinc sites. All distances lie within expected ranges.[1]

Structural Site	(Å)	Sensory Site	(Å)
Zn-S Cys-83	2.34 ± 0.02	Zn-OD1 Asp-77	2.34 ± 0.04
Zn-S Cys-86	2.29 ± 0.02	Zn-OD2 Asp-77	2.02 ± 0.01
Zn-S Cys-123	2.33 ± 0.03	Zn-N His-79	2.02 ± 0.05
Zn-S Cys-126	2.33 ± 0.01	Zn-S Cys-101	2.36 ± 0.04
		Zn-N His-115	1.95 + 0.05

Supplementary Table 4| Genes with promoters that harbour two or more Zur boxes in a range of bacterial genomes. Pairs of Zur boxes are present in the promoters of genes for uptake (*znuABC* components and their orthologues; shaded in dark orange) and other genes associated with zinc scarcity (COG0523 genes, ribosomal proteins; *folE* and *hemB*; shaded in rose). There are only four examples where a pair of boxes is associated with zinc excess, one of which is *Syn. sp. WH8102 bmtA* (highlighted in blue). For unshaded entries, associations are unknown, and we have also not shaded autoregulated *zur*. Entries have been extracted from the RegPrecise database[2], and associations with zinc status are based on reference 3.

Species	Zur box 1	Zur box 2	gene
Bifidobacterium animalis subsp. lactis AD011	-60	-92	zinT
Corynebacterium glutamicum ATCC 13032	-60	-105	FAD-dependent pyridine nucleotide-disulphide oxidoreductase
Corynebacterium glutamicum ATCC 13032	4	-40	mntA
Corynebacterium glutamicum ATCC 13032	-19	-25	DIP0441 putative ABC transport system, permease protein
Corynebacterium glutamicum ATCC 13032	-34	-79	yciC
Corynebacterium diphtheriae NCTC 13129	-30	-68	sapD putative surface-anchored protein
Corynebacterium aurimucosum ATCC 700975	-67	-116	cmrA
Corynebacterium jeikeium K411	-42	-75	sapD
Corynebacterium urealyticum DSM 7109	-103	-160	sapD
Saccharopolyspora erythraea NRRL 2338	-34	-88	rpmB2
Saccharopolyspora erythraea NRRL 2338	-11	-65	yciC
Nocardioides sp. JS614	-8	-30	znuA
Arthrobacter sp. FB24	-55	-98	znuA
Brachybacterium faecium DSM 4810	-17	-62	AAur_1965 WD40/YVTN repeat-like-containing domain
Janibacter sp. HTCC2649	-17	-39	znuA
Kocuria rhizophila DC2201	-63	-100	znuA
Renibacterium salmoninarum ATCC 33209	-62	-103	znuA
Mycobacterium smegmatis str. MC2 155	-44	-90	znuA
Mycobacterium smegmatis str. MC2 155	-30	-76	yciC2
Mycobacterium tuberculosis H37Rv	-30	-85	znuA
Mycobacterium tuberculosis H37Rv	-45	-99	rpmB1
Mycobacterium tuberculosis H37Rv	-48	-103	rpmB2
Mycobacterium tuberculosis H37Rv	-31	-85	yciC
Mycobacterium abscessus ATCC 19977	-19	-25	yciC2
Mycobacterium abscessus ATCC 19977	-51	-106	yciC
Mycobacterium abscessus ATCC 19977	-35	-90	rpmB1
Mycobacterium avium 104	-65	-71	ppe3
Mycobacterium avium 104	-63	-69	ppe3
Mycobacterium avium 104	-38	-93	rpmB1
Mycobacterium avium 104	-81	-136	yciC3
Mycobacterium marinum M	-43	-99	rpmB1
Mycobacterium marinum M	-51	-107	yciC
Mycobacterium flavescens PYR-GCK	-44	-99	rpmB1
Mycobacterium flavescens PYR-GCK	-32	-87	yciC3
Mycobacterium sp. JLS	-34	-89	rpmB1
Mycobacterium sp. JLS	-33	-88	yciC
Mycobacterium vanbaalenii PYR-1	-25	-81	znuA
Mycobacterium vanbaalenii PYR-1	-33	-88	rpmB1
Mycobacterium vanbaalenii PYR-1	-35	-90	yciC3
Nocardia farcinica IFM 10152	-40	-95	rpmB2
Nocardia farcinica IFM 10152	-33	-88	yciC
Rhodococcus erythropolis PR4	-41	-95	rpmB2
Rhodococcus erythropolis PR4	-30	-84	yciC
Rhodococcus sp. RHA1	-63	-117	rpmB2
Rhodococcus sp. RHA1	-31	-47	znuB2

Rhodococcus sp. RHA1	-29	-83	yciC
Rhodococcus opacus B4	-62	-116	rpmB2
Rhodococcus opacus B4	-31	-47	znuB2
Streptomyces scabiei 87.22	-17	-39	znuA
Streptomyces griseus subsp. griseus NBRC 13350	-17	-39	znuA
Streptomyces coelicolor A3(2)	-17	-39	znuA
Streptomyces avermitilis MA-4680	-17	-39	znuA
Chloroflexus aggregans DSM 9485	-20	-79	yciC
Herpetosiphon aurantiacus ATCC 23779	-20	-82	Rcas_3893 hypothetical protein
Cyanothece sp. ATCC 51142	127	81	thrS2
Cyanothece sp. ATCC 51142	-52	80	yciC
Cyanothece sp. ATCC 51142	-32	-78	hemB2
Cyanothece sp. ATCC 51142	-46	-72	hemB2
Cyanothece sp. ATCC 51142	-36	-62	PF07992 Predicted FAD-dependent oxidoreductase
Cyanothece sp. ATCC 51142	-38	-77	yciC2
Cyanothece sp. ATCC 51142	-46	-72	znuA
Cyanothece sp. PCC 8801	-32	-72	hemB2
Cyanothece sp. PCC 8802	-38	-76	yciC2
Nostoc sp. PCC 7120 [b)]	-80	-110	znuA
Nostoc sp. PCC 7120	-23	-29	alr1197 Putative metallochaperone
Nostoc sp. PCC 7120	-260	-266	alr3242 Zinc-regulated TonB-dependent outer membrane receptor
Synechococcus sp. WH 8102	-79	-105	bmtA
Bacillus subtilis subsp. subtilis str. 168	-52	-92	adcA
Bacillus amyloliquefaciens FZB42	-42	-105	adcA
Bacillus amyloliquefaciens FZB42	-38	-103	yciC
Bacillus pumilus SAFR-032	-151	-188	ytiB Carbonic anhydrase
Bacillus licheniformis DSM 13	-41	-245	NADH-ubiquinone oxidoreductase 51 kDa subunit
Bacillus licheniformis DSM 13	-44	-120	folE2 GTP cyclohydrolase I
Bacillus cereus ATCC 14579	-39	-93	yciC
Bacillus halodurans C-125	-54	-195	yciC
*Paenibacillus sp. JDR-2** [a)]	-35	-67	adcC
Oceanobacillus iheyensis HTE831	-41	-57	zinT
Oceanobacillus iheyensis HTE831	-40	-55	OB3434 Hypothetical protein
Oceanobacillus iheyensis HTE831	-277	-294	znuC
Clostridium acetobutylicum ATCC 824	-111	-193	rpsD2
Clostridium butyricum 5521	-95	-101	rpsD2
Clostridium beijerincki NCIMB 8052	-112	-168	feoA ferrous iron transport protein A
Clostridium novyi NT	-34	-40	fld Flavodoxin
Clostridium novyi NT	-42	-175	znuA2
Clostridium perfringens ATCC 13124	-54	-121	fld Flavodoxin
Clostridium tetani E88	-40	-105	znuA
Clostridium tetani E88 [a)]	-38	-89	znuC
Enterococcus faecalis V583	-59	-215	fhuC Ferrichrome transport ATP-binding protein
Enterococcus faecalis V583	-40	-196	fhuD Ferrichrome-binding periplasmic protein precursor
Enterococcus faecium DO [a)]	-87	-93	Heavy metal translocating P-type ATPase
Lactobacillus casei ATCC 334	-40	-183	znuA
Lactobacillus casei ATCC 334	-40	-183	lp_0947 Conserved hypothetical protein
Lactobacillus plantarum WCFS1	-39	-88	znuA
Lactobacillus rhamnosus GG	-40	-181	znuA
Lactobacillus salivarius subsp. salivarius UCC118	-42	-63	rpsN2
Leuconostoc mesenteroides subsp. mesenteroides ATCC 8293	-142	-171	znuA

Leuconostoc mesenteroides subsp. mesenteroides ATCC 8293	-43	-65	lp_0947 Conserved hypothetical protein
Oenococcus oeni PSU-1	-76	-118	znuA
Oenococcus oeni PSU-1	-53	-95	zur
Listeria monocytogenes EGD-e	-34	-97	ZnuA
Listeria monocytogenes EGD-e	-37	-69	ZnuA2
Listeria seeligeri serovar 1/2b str. SLCC3954	-32	-96	znuA
Listeria seeligeri serovar 1/2b str. SLCC3954	-37	-69	ZnuA2
Listeria welshimeri serovar 6b str. SLCC5334	-34	-97	ZnuA
Listeria welshimeri serovar 6b str. SLCC5334	-37	-69	ZnuA2
Listeria innocua Clip11262	-34	-97	ZnuA
Listeria innocua Clip11262	-37	-69	ZnuA2
Staphylococcus epidermidis ATCC 12228	-35	-226	znuA2
Staphylococcus epidermidis ATCC 12228	-53	-244	SA2370 hypothetical protein
Staphylococcus haemolyticus JCSC1435	-35	-188	znuA2
Staphylococcus haemolyticus JCSC1435	-101	-254	SA2368 hypothetical protein
Macrococcus caseolyticus JCSC5402	-35	-168	znuA
Agrobacterium tumefaciens str. C58 (Cereon) [a)]	-61	-91	ZnuC
Agrobacterium tumefaciens str. C58 (Cereon)	-120	-262	yciC2
Burkholderia pseudomallei K96243	-87	-199	omr Predicted zinc-related TonB-dependent outer membrane transporter
Burkholderia mallei ATCC 23344	-87	-199	omr Predicted zinc-related TonB-dependent outer membrane transporter
Burkholderia vietnamiensis G4	-74	-179	omr Predicted zinc-related TonB-dependent outer membrane transporter
Polaromonas naphthalenivorans CJ2	-45	-77	zur
Polaromonas naphthalenivorans CJ2	-219	-251	znuC
Rhodoferax ferrireducens DSM 15236	-55	-81	zur
Rhodoferax ferrireducens DSM 15236	-251	-277	znuC
Ralstonia metallidurans CH34	-71	-99	yciC2
Nitrosomonas europaea ATCC 19718	-193	-233	omr2 Predicted zinc-related TonB-dependent outer membrane transporter
Methylobacillus flagellatus KT	-34	-72	dksA2 DnaK suppressor protein
Pseudoalteromonas atlantica T6c	-32	-62	COG4525 ABC transporter ATP-binding protein
Pseudoalteromonas atlantica T6c	-175	-204	zbp Predicted zinc-binding protein
Pseudoalteromonas atlantica T6c	-36	-65	omr1 Zinc-regulated outer membrane receptor
Alteromonas macleodii 'Deep ecotype'	-50	-55	COG4525 ABC transporter ATP-binding protein
Colwellia psychrerythraea 34H	-169	-194	omr1 Zinc-regulated outer membrane receptor
Alteromonadales bacterium TW-7	-118	-146	zur2 Zinc uptake regulation protein paralog
Alteromonadales bacterium TW-7	-36	-64	omr1 Zinc-regulated outer membrane receptor
Alteromonadales bacterium TW-7	-47	-85	omr Zinc-regulated outer membrane receptor
Pseudoalteromonas haloplanktis TAC125	-48	-86	omr Zinc-regulated outer membrane receptor
Pseudoalteromonas tunicata D2	-43	-83	omr Zinc-regulated outer membrane receptor
Serratia proteamaculans 568	-80	-85	omr1 predicted zinc-related TonB-dependent outer membrane transporter
Hahella chejuensis KCTC 2396	-206	-211	zur2
Oceanobacter sp. RED65	-47	-52	PF08856 hypothetical protein
Oceanospirillum sp. MED92	-49	-152	pyrC2 Dihydroorotase
Oceanospirillum sp. MED92	-35	-138	ribosomal protein, L31P family protein
Oceanospirillum sp. MED92	-44	-49	PF08856 hypothetical protein
Marinomonas sp. MWYL1	-18	-23	PF08856 hypothetical protein
Teredinibacter turnerae T7901	-114	-119	yciC
Reinekea sp. MED297	-119	-124	znuC

Alcanivorax borkumensis SK2	-51	-56	hypothetical protein
Pseudomonas entomophila L48	-65	-70	yciC
Psychromonas ingrahamii 37	-52	-91	zip
Moritella sp. PE36	-199	-239	ZnuC
Moritella sp. PE36	-52	-91	zip
Vibrio harveyi ATCC BAA-1116	-36	-70	omr predicted zinc-related TonB-dependent outer membrane transporter
Vibrio parahaemolyticus RIMD 2210633	-33	-62	omr predicted zinc-related TonB-dependent outer membrane transporter
Vibrio angustum S14	-109	-133	znuA
Vibrio angustum S14	-59	-83	znuC
Petrotoga mobilis SJ95	-80	-97	zur

[a)] There are further Zur boxes in these promoters

[b)] *Nostoc spp*. are also known as *Anabaena spp*.

Supplementary Table 5| Distribution of *bmtA* and putative Zur-regulated *znuA* genes in marine *Synechococcus* strains. Information was extracted from Cyanorak[4]; the cluster numbers refer to this database. Upstream regions of genes were screened for regulatory sequences using the FIMO tool (part of the MEME suite)[7]. *Prochlorococcus* genomes were also searched, but *bmtA* genes are absent in all sequenced *Prochlorococcus* strains. Cyanorak lists four different *bmtA* clusters; these are shown with symbols: CK_00049937 (1), CK_00042830 (2), CK_00057549 (3) and CK_00053974 (4). Metallothionein genes with predicted Zur boxes in their upstream region are marked with superscript ^{Zur}. As previously reported[5,6], all *Synechococcus* and *Prochlorococcus* strains contain predicted *znuABC* systems; these are collected in Cyanorak clusters CK_00001600 (of which SYNW0971 is a member) and CK_00002462 (of which SYNW2481 is a member). *ZnuA* upstream regions were screened for Zur boxes, which are shown as (I) for CK_00001600 and as (II) for CK_00002462. CK_00001600 was found in all *Prochlorococcus* strains and all except 7 (MIT9201(HLII), MIT0601(LLIII), MIT9211(LLIII), MIT0701(LLIV), MIT0702(LLIV), MIT0703 (LLIV) and MIT9303(LLIV)) contained predicted Zur boxes; CK_00002462 was found only in MIT9303, and a Zur box was identified upstream of it. Cluster CK_00001600 was found in all *Synechococcus* strains except MINOS11 (5.3) and RCC307 (5.3) and all *Cyanobium* sp (5.2). Cluster CK_00002462 was found in *Cyanobium* sp (5.2) and *Synechococcus* sp. CC9311(Ia), WH8020(Ia), MVIR-18-1(Ib), ROS8604(Ib), SYN20(Ib), A15-24(IIIa), A18-40(IIIa), A18-46.1(IIIa), BOUM118(IIIa), RS9915(IIIa), WH8102(IIIa), WH8103(IIIa), A15-28(IIIb), RS9909(VIII), RS9917(VIII), WH8101(VIII), BIOS-U3-1(CRD1a), MITS9220(CRD1a), BIOS-E4-1(CRD1b), CC9616(EnvC). Entries highlighted in yellow refer to *bmtA* promoters with two predicted Zur boxes (see Supplementary Table 5).

**Species**	**Subclade**	***bmtA***	*znuA* with Zur boxes
Synechococcus sp. CC9311	Ia	1^{Zur}, 1, 2, 4	
Synechococcus sp. WH8020	Ia	1^{Zur}	
Synechococcus sp. MVIR-18-1	Ib	2	
Synechococcus sp. PROS-9-1	Ib	2	I
Synechococcus sp. ROS8604	Ib	2	II
Synechococcus sp. SYN20	Ib	2	
Synechococcus sp. WH8016	Ib	2	I
Synechococcus sp. A15-44	IIa	1^{Zur}	I
Synechococcus sp. KORDI-52	IIa	1	
Synechococcus sp. M16.1	IIa	1	I
Synechococcus sp. RS9902	IIa	1^{Zur}	I
Synechococcus sp. RS9907	IIa	1^{Zur}	I
Synechococcus sp. TAK9802	IIa	1, 2, 2	I
Synechococcus sp. WH8109	IIa	1^{Zur}	I
Synechococcus sp. A15-62	IIc	1^{Zur}	I
Synechococcus sp. CC9605	IIc	1^{Zur}	
Synechococcus sp. PROS-U-1	IIh	1^{Zur}, 2	
Synechococcus sp. A15-24	IIIa	1^{Zur}	I, II
Synechococcus sp. A18-40	IIIa	1^{Zur}	I
Synechococcus sp. A18-46.1	IIIa	1^{Zur}	I
Synechococcus sp. BOUM118	IIIa	1^{Zur}	I
Synechococcus sp. RS9915	IIIa	1^{Zur}	I
Synechococcus sp. WH8102	IIIa	1^{Zur}	I
Synechococcus sp. WH8103	IIIa	1^{Zur}	I
Synechococcus sp. A15-28	IIIb	1^{Zur}	I
Synechococcus sp. BL107	IVa		I
Synechococcus sp. CC9902	IVa		
Synechococcus sp. BMK-MC-1	V	1	I
Synechococcus sp. WH7803	V	1	I
Synechococcus sp. MEDNS5	VIa	1	I
Synechococcus sp. WH7805	VIa		I
Synechococcus sp. PROS-7-1	VIb	1	I
Synechococcus sp. A18-25c	VIIa	1	
Synechococcus sp. A15-60	VIIa		

Synechococcus sp. NOUM97013	VIIb	1[Zur]	
Synechococcus sp. RS9909	VIII		
Synechococcus sp. RS9917	VIII		
Synechococcus sp. WH8101	VIII		I
Synechococcus sp. RS9916	IX		I
Synechococcus sp. BIOS-U3-1	CRD1a	1[Zur], 2, 3	
Synechococcus sp. MITS9220	CRD1a	1[Zur], 1, 2	I, II
Synechococcus sp. BIOS-E4-1	CRD1b	1[Zur], 1, 1, 1, 2, 2, 3	II
Synechococcus sp. CC9616	EnvC	1	II
Synechococcus sp. KORDI-100	UC-A	1[Zur]	I
Synechococcus sp. A15-127	WPC1	1, 2	I
Synechococcus sp. KORDI-49	WPC1	1	I
Synechococcus sp. CB0101	5.2A		
Synechococcus sp. CB0205	5.2A		
Cyanobium sp. NS01	5.2B	1	
Cyanobium sp. PCC6307	5.2B	1	II
Cyanobium sp. PCC7001	5.2B	1	
Synechococcus sp. WH5701	5.2B	1, 1	
Synechococcus sp. MINOS11	5.3		
Synechococcus sp. RCC307	5.3		

Supplementary Table 6| Predicted Zur boxes in cyanobacterial metallothionein promoters. Promoters were analysed using the FIMO tool (part of the MEME suite)[7] with default parameters. The motif (NTNANAATGATNATCATTNTNAN) was inferred from the RegPrecise database[8] for cyanobacterial Zur boxes. Rows are alternately shaded to facilitate identification of promoters with two Zur boxes. Presence of two or more Zur boxes is highlighted in red in column 3. Shading in the final column highlights conservation.

Species (*Synechococcus* sp.)	Subclade	Sequence Name	Start	End	Matched Sequence ntnanaatgatnatcattntnan
CC9311	Ia	CK_Syn_CC9311_01081	120	142	ctaagaatgataattgtttttat
CC9311	Ia	CK_Syn_CC9311_01081	87	109	gtgaaaatgattcccatttcttg
WH8020	Ia	CK_Syn_WH8020_50068	87	109	gtgagaatgattaccgtttctag
WH8020	Ia	CK_Syn_WH8020_50068	119	141	ttcaaaacgataattgtattaat
A15-44	IIa	CK_Syn_A15-44_01892	85	107	aaaatattgataacgattctcat
RS9902	IIa	CK_Syn_RS9902_01335	84	106	aaaagattgacaatgattctcac
RS9907	IIa	CK_Syn_RS9907_01770	85	107	gaaatattgataacgattctcac
WH8109	IIa	CK_Syn_WH8109_50021	83	105	gaaagattgataataattctcac
A15-62	IIc	CK_Syn_A15-62_01644	85	107	gaaaaattgatagtgattctcac
CC9605	IIc	CK_Syn_CC9605_02737	83	105	gaaagattgataataattctcac
PROS-U-1	IIh	CK_Syn_PROS-U-1_01885	92	114	gcgagaatgattatcactttaag
A15-24	IIIa	CK_Syn_A15-24_50004	81	103	caaagaatgattattattgtcag
A15-24	IIIa	CK_Syn_A15-24_50004	56	78	ataagaaagagaatcattattgg
BOUM118	IIIa	CK_Syn_BOUM118_50003	84	106	cattgaatgataatcattatcag
BOUM118	IIIa	CK_Syn_BOUM118_50003	58	80	ataagaatgggaatcattgtttg
BOUM118	IIIa	CK_Syn_BOUM118_50003	90	112	atgataatcattatcagtgatct
RS9915	IIIa	CK_Syn_RS9915_50003	81	103	tattgaatgataatcattgtcag
RS9915	IIIa	CK_Syn_RS9915_50003	56	78	ataagaacgagaatcattatcgg
WH8102	IIIa	CK_Syn_WH8102_00359	83	105	aaaagaatgataatcattatcgg
WH8102	IIIa	CK_Syn_WH8102_00359	57	79	ataagaataattctcattattgg
WH8103	IIIa	CK_Syn_WH8103_50030	83	105	aaaagaatgataatcattatcgg
WH8103	IIIa	CK_Syn_WH8103_50030	57	79	ataagaataattctcattattgg
A18-40	IIIa	CK_Syn_A18-40_50004	80	102	tattgaatgataatcattgtcag
A18-40	IIIa	CK_Syn_A18-40_50004	55	77	ataagaacgagaatcattatcgg
A18-46.1	IIIa	CK_Syn_A18-46.1_50004	56	78	ataagaatgagaatcattattgg
A15-28	IIIb	CK_Syn_A15-28_50003	78	100	caaagaatgattattattatcag
A15-28	IIIb	CK_Syn_A15-28_50003	53	75	ataagaaagagaatcattattgg
NOUM97013	VIIb	CK_Syn_NOUM97013_01126	74	96	caaaacacgagaatcattctcat
BIOS-U3-1	CRD1a	CK_Syn_BIOS-U3-1_50004	91	113	atgaaaatgataatcgttttatc
MITS9220	CRD1a	CK_Syn_MITS9220_50005	123	145	ttgaaaacgattatcacgctggt
BIOS-E4-1	CRD1b	CK_Syn_BIOS-E4-1_50003	86	108	gtgacaacgattatcgtttttgt
BIOS-E4-1	CRD1b	CK_Syn_BIOS-E4-1_50003	118	140	ttgaaaacgataatcactatctt
KORDI-100	UC-A	CK_Syn_KORDI-100_50023	92	114	aagggaatgattctcattctttt

Supplementary Table 7| *Escherichia coli* strains used in this work.

Strain	Description	Supplier
S17-1 λPir[9]	TpR SmR *recA*, *thi*, *pro*, *hsd*R-M+RP4: 2-Tc:Mu: Km Tn7 λpir	Dr. Joseph Christie-Oleza
BL21 (DE3) pLysS	F– *ompT* hsdSB(rB⁻, mB⁻) *gal dcm rne131* (DE3) pLysS (Cm^R)	Novagen
DH5-α	F– Φ80lacZΔM15 Δ(lacZYA-argF) U169 *recA1 endA1 hsdR17* (r_K^-, m_K^+) *phoA supE44* λ– thi-1 *gyrA96 relA1*	Invitrogen
TEV system[10]	*E. coli* BL21 (DE3) CodonPlus-RIL containing the TEV protease expression vector pRK793	Dr. Göran M. Rashid

Supplementary Table 8| Composition of ASW medium without zinc (ASW_{-Zn}).*)

Macronutrient	Final concentration
NaCl	428 mM (25 g×L^{-1})
$MgCl_2$•$6H_2O$	10 mM (2 g×L^{-1})
KCl	6.7 mM (0.5 g×L^{-1})
$NaNO_3$	8.8 mM (0.75 g×L^{-1})
K_2HPO_4•$3H_2O$	0.172 mM (3*10^{-2} g×L^{-1})
$MgSO_4$•$7H_2O$	14 mM (3.5 g×L^{-1})
$CaCl_2$•$2H_2O$	3.4 mM (0.5 g×L^{-1})
Tris HCl buffer	9.08 mM (1.1 g×L^{-1})
**Trace metals**	**Final concentration**
H_3BO_3	46.1µM (2.86*10^{-3} g×L^{-1})
$MnCl_2$•$4H_2O$	9.1 µM (1.81*10^{-3} g×L^{-1})
Na_2MoO_4•$2H_2O$	1.8 µM (4.0*10^{-4} g×L^{-1})
$CuSO_4$*$5H_2O$	0.032 µM (8.0*10^{-6} g×L^{-1})
$Co(NO_3)_2$•$6H_2O$	0.17 µM (5.0*10^{-5} g×L^{-1})
$FeCl_3$•$7H_2O$	10.4 µM (3.0*10^{-3} g×L^{-1})
EDTA (Na_2Mg)	1.4 µM (5.0*10^{-4} g×L^{-1})

*) The original concentration of $ZnSO_4$•$7H_2O$ in ASW was given as 0.772 µM (2.2*10^{-4} g×L^{-1})[11]

Supplementary Table 9| Plasmids used in this work.

Plasmid	Description	Supplier
PGP704CmKm[9]	Ap^r Km^r Cm^r; pGP704-derivative containing the Km^r and Cm^r gene at the *Eco*RI site of its MCS	Dr. Christie-Oleza
pGP704CmKm Zur	pGP704CmKm-derivative containing the *zur* (21-314) insert at the *Xba*I and *Sal*I sites of its MCS	This study
pET155/D-topo Zur[12]	pET155/D-topo derivative containing synthetic *zur* insert cloned into the TOPO-cloning site	Dr. Amira Ksibe

Supplementary Table 10| PCR primers used in this study. Restriction sites are highlighted**.**

**Name**	**Sequence**	**Restriction site**
Zur_F	ATGTCGACTTTGAACGCCCGTCAACAGG	*Sal*I
Zur_Re	ATTCTAGAGTCAGCAGGCACGTCGATG	*Xba*I
A_F	CTGGCCAATGTGATGTTC	
B_Re	TTTCATCGCTCTGGAGTG	
C_Re	GCAGGAGATCAAGACTTTCG	
D_F	CGTCAACACGGGATAATACC	
16S_27F	AGAGTTTGATCCTGGCTCAG	
16S_1492Re	ACCTTGTTACGACTT	
ZnuA_F	AGCAAGGAGATCCAATCTTCAG	
ZnuA_Re	GGCAATCCGGTTGACTTACT	
BmtA_F	GCGCAATTGTTCTTCAAGGTAA	
BmtA_Re	TCCGCACTTACATCCACAAG	
pepC_F	CGCAGTGATCTGGAACTGAT	
pepC_Re	CGAAGATGTGGACCTGATTGA	
pznuABC_F	GAGATTCCTGGCTGCTAGATG	
pznuABC_Re	GTTCCATCCACAGCCACAAC	

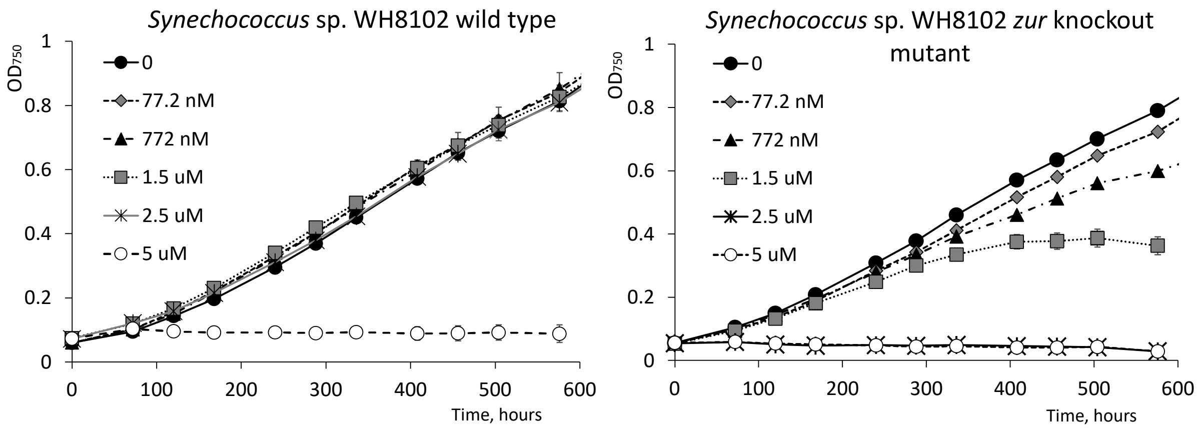

Supplementary Figure 1. Growth curves of wild-type and *zur* mutant *Synechococcus* sp. WH8102 at different zinc concentrations added to zinc-depleted ASW medium. Specific growth rates are shown in Figure 1c. Data points represent the means ± standard deviation from n = 3 independent biological replicates.

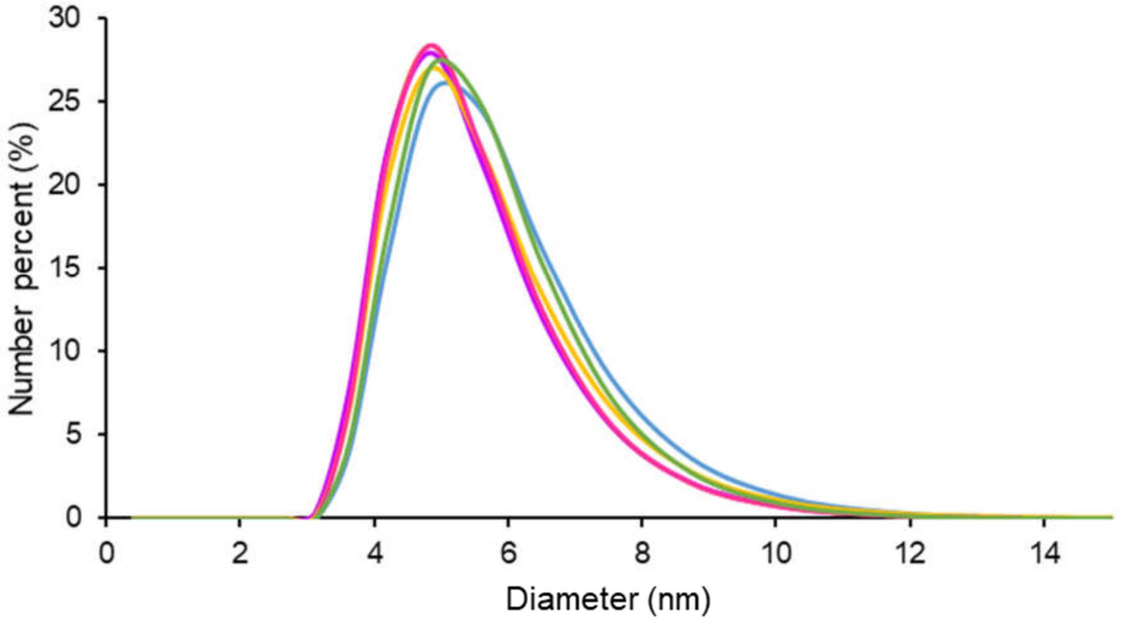

Entity	Hydrodynamic diameter
**Experimental for Zn_2SynZur**	**5.39±0.15**
Monomers (A-D)	4.47±0.04
**Dimers (AC and CD)**	**5.20±0.09**
2 dimers in asymm. unit	6.79
Back-to-back tetramer	7.59
Intertwined tetramer (5nhk)	6.30

Supplementary Fig. 2. Estimation of oligomerisation state of Zn_2SynZur by dynamic light scattering. a, The hydrodynamic diameter number distribution of 20 µM SynZur in 50 mM Tris as measured by dynamic light scattering (DLS). **b,** Comparison of experimental and calculated hydrodynamic diameter of Zn_2SynZur. The uncertainty in the experimental data corresponds to the standard deviation from n = 6 repeated measurements. The theoretical data are the mean ± standard deviation from either 4 monomers of 2 dimers in the asymmetric unit. The DLS data are consistent with a dimer dominating in solution. Theoretical hydrodynamic diameters are based on the X-ray crystal structure determined in this work.

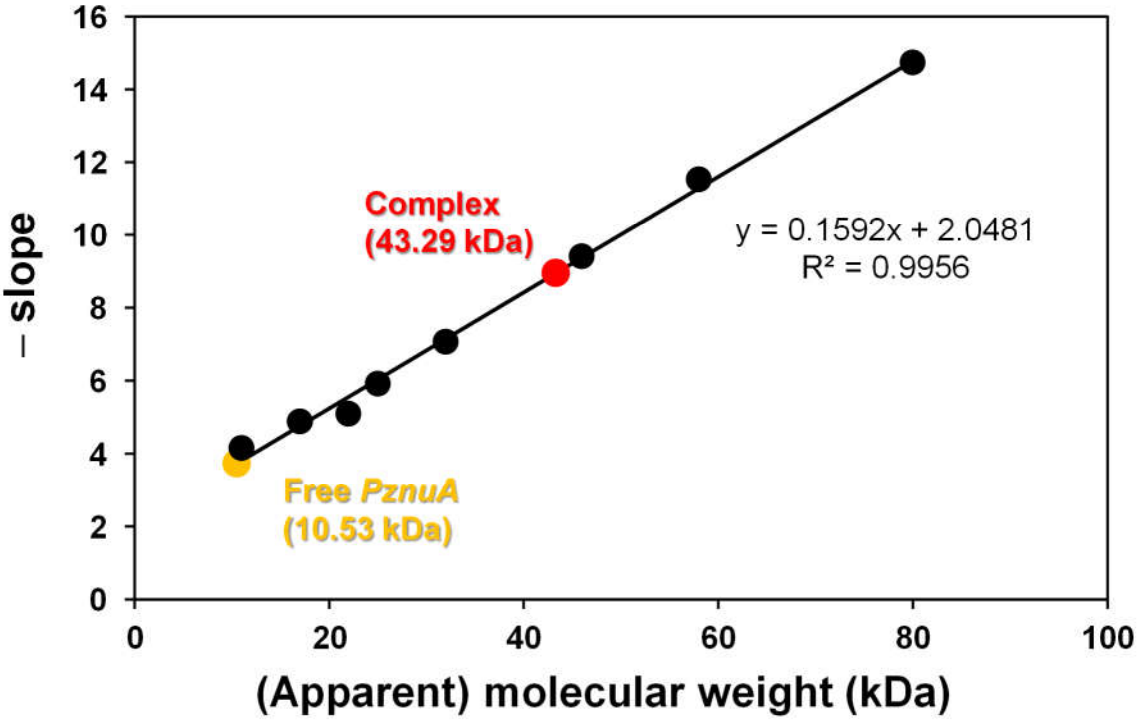

Supplementary Fig. 3. Ferguson plot analysis of SynZur binding to the *PznuA* promoter. Negative slopes for standards (P77125 New England Biolabs Color Prestained Protein Standard, Broad Range), free DNA, and the *PznuA*-SynZur complex were derived from running 8% and 12% gels, and plotting 100×log(Rf×100; Rf = mobility relative to low molecular weight standard; MyTaq Red) in dependence of gel percentage. The difference between the apparent masses of the free promoter and the complex with SynZur is 32.76 kDa, close to that expected for a SynZur dimer (31.1 kDa).

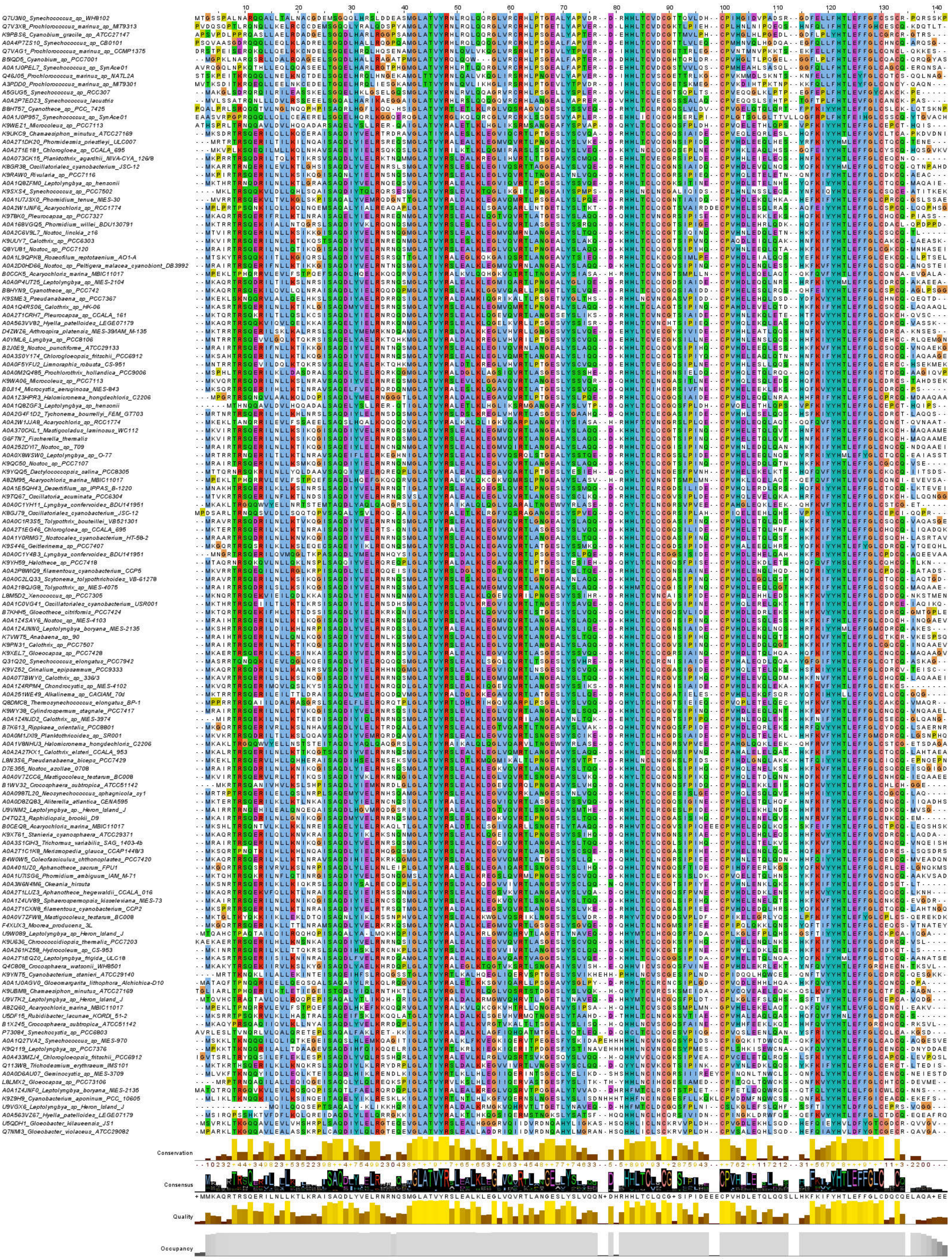

Supplementary Fig. 4| The four residues of the new sensory site are conserved in putative cyanobacterial Zur proteins. Multiple sequence alignment of putative cyanobacterial Zur proteins retrieved via a BLAST search from UniProtKB reference proteomes+SwissProt. Note that these databases do not include many marine *Prochlorococcus* proteomes, but inspection of a previously published alignment[13] confirms the high degree of conservation of the four

zinc-binding residues also in other *Prochlorococcus* strains (and the absence of C95 and H115 in other Fur family proteins). N- and C-terminal extensions were trimmed for clarity, and annotations were shortened to contain accession code and strain name. Alignment was carried out in MEGA6 using MUSCLE. The display was generated using Jalview 2.11.1.3.

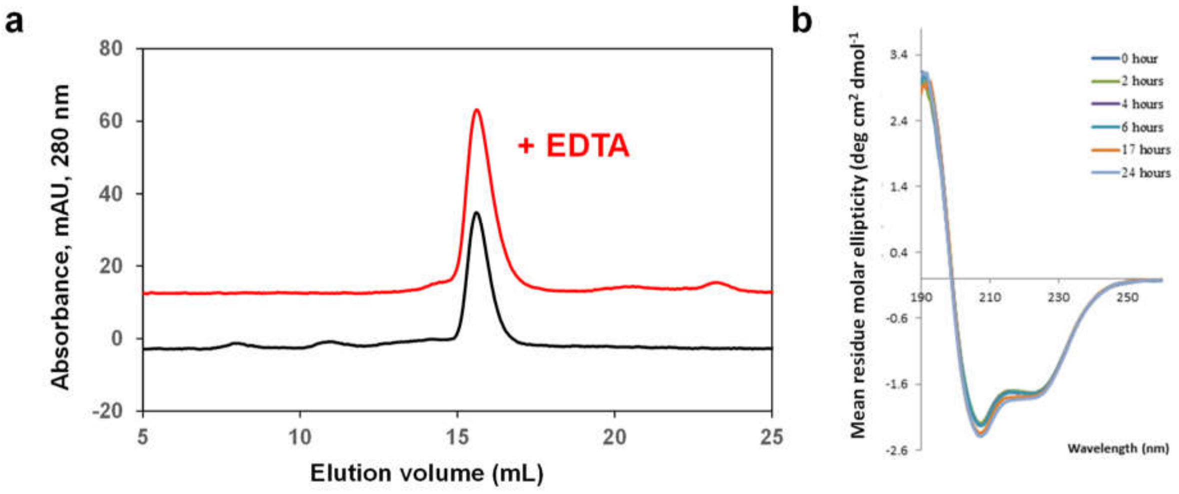

c

Analysis	% α helices (as purified)	% α helices (after EDTA treatment)	% β strands (as purified)	% β strands (after EDTA treatment)	% Other (as purified)	% Other (after EDTA treatment)
SELCON	35	37.2	33	29.5	22.5	23
CONTIN	24	24	35	35	41	41
RAUSSENS	45	43	17.5	19	34.2	35

Supplementary Fig. 5| SEC and circular dichroism (CD) spectroscopic analysis of SynZur in the presence of EDTA. **a,** Comparison of SEC traces in the presence and absence of 1 mM EDTA in the elution buffer (40 μM protein; 20 mM ammonium bicarbonate, 150 mM NaCl; pH 8). EDTA did not elicit changes in oligomerisation state or perceptible changes in shape. Further SEC experiments conducted at lower concentrations (30, 30, 10 and 5 μM), and in the presence of dithiothreitol (DTT, to exclude the possibility of oxidative crosslinking) also did not reveal any changes in shape or oligomerisation state (data not shown). **b,** CD spectra of SynZur (25 μM in 5 mM Tris-Cl pH 8) before and after reaction with a 10-fold excess of EDTA. **c,** Analysis of secondary structure content using 3 different programs, comparing results of untreated SynZur with SynZur after 24 hours of EDTA treatment. Like observed for other Fur-family proteins, secondary structure is not significantly affected by loss of the sensory metal. The small differences in molar ellipticity values between different timepoints in (b) are likely due to a small degree of solvent evaporation over the prolonged periods for this experiment. The analysis in (c) clearly indicates that these are not related to even subtle changes in secondary structure.

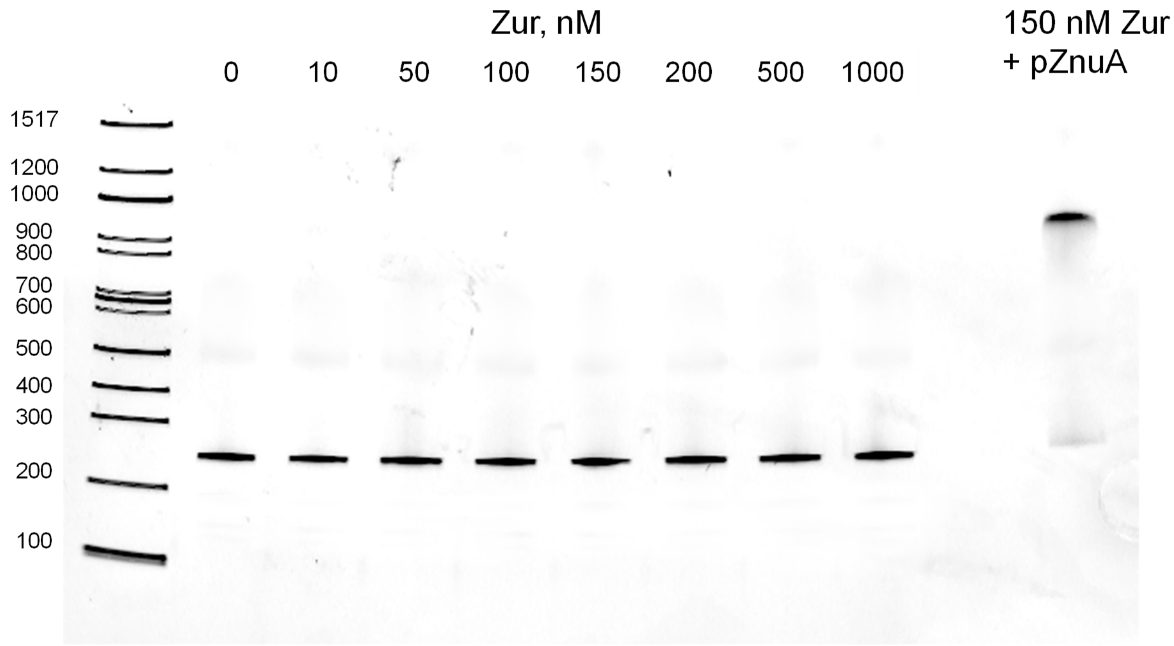

Supplementary Fig. 6| SynZur does not bind to the *zur* (synw_2401) promoter region (220 bps). All samples contained 5 ng DNA probe. Experimental conditions are described in Methods (DNA-binding experiments).

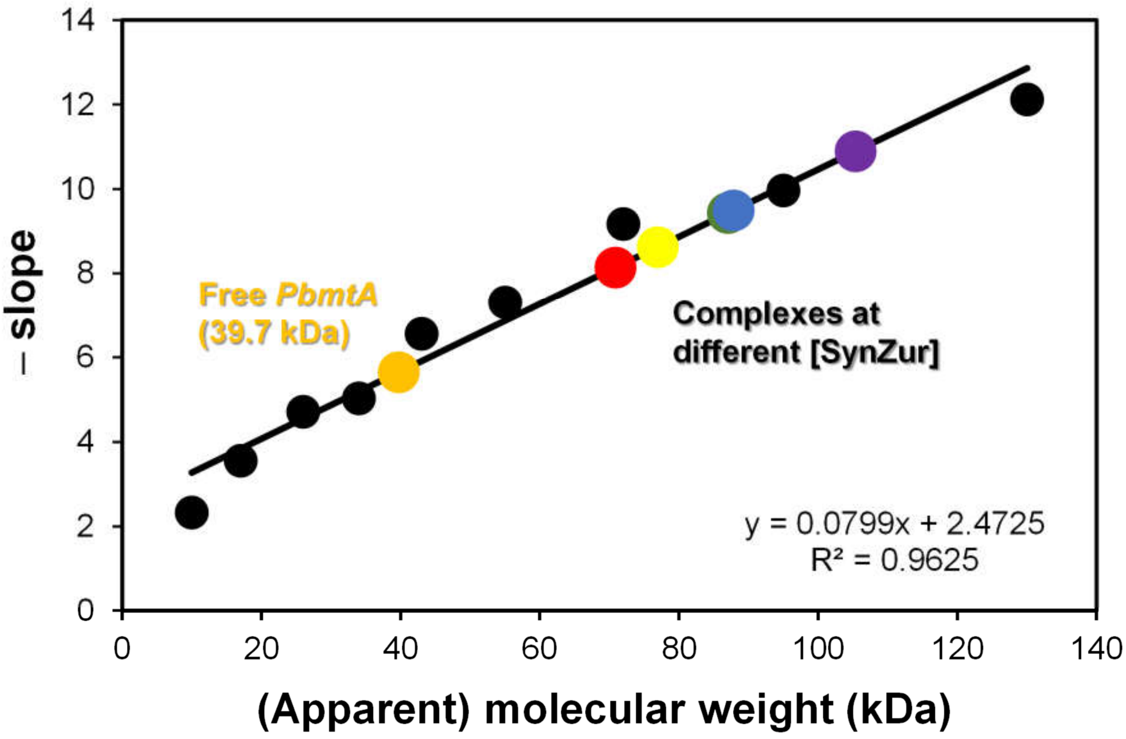

[SynZur]	Apparent molecular weight	Corresponding number of dimers
50 nM	31.15	1.00±0.07
100 nM	37.22	1.20±0.09
500 nM	47.31	1.53±0.11
1 µM	48.12	1.55±0.12
5 µM	65.64	2.12±0.16

Supplementary Fig. 7| Ferguson plot analysis of SynZur binding to the *PbmtA* promoter. Negative slopes for standards (P7719S New England Biolabs Color Prestained Protein Standard, Broad Range), free DNA, and the *PbmtA*-SynZur complex were derived from running 6%, 7% and 8% gels, and plotting 100×log(Rf×100; Rf = mobility relative to low molecular weight standard; MyTaq Red) in dependence of gel percentage. Apparent molecular weights are given after subtraction of the apparent molecular weight of the *PbmtA* promoter DNA.

References for Supplementary Information

1. Harding, M. M. Geometry of metal-ligand interactions in proteins. *Acta Cryst. D* **57**, 401-411 (2000).
2. Novichkov, P. S. *et al.* RegPrecise 3.0 - A resource for genome-scale exploration of transcriptional regulation in bacteria. *BMC Genomics* **14**, 745 (2013).
3. Mikhaylina, A., Ksibe, A. Z., Scanlan, D. J. & Blindauer, C. A. Bacterial zinc uptake regulator proteins and their regulons. *Biochem. Soc. Trans.* **46**, 983–1001 (2018).
4. Garczarek, L. *et al.* Cyanorak v2.1: a scalable information system dedicated to the visualization and expert curation of marine and brackish picocyanobacteria genomes. *Nucleic Acids Res.* **1–10** (2020).
5. Blindauer, C. A. Zinc-handling in cyanobacteria: An update. *Chem. Biodivers.* **5**, 1990–2013 (2008).
6. Scanlan, D. J. *et al.* Ecological genomics of marine picocyanobacteria. *Microbiol. Mol. Biol. Rev.* **73**, 249–299 (2009).
7. Grant C. E., Bailey, T. L., Noble W. S., "FIMO: Scanning for occurrences of a given motif", *Bioinformatics* **27**, 1017–1018 (2011).
8. Novichkov, P. S. *et al.* RegPrecise 3.0 - A resource for genome-scale exploration of transcriptional regulation in bacteria. *BMC Genomics* **14**, 745 (2013).
9. Christie-Oleza, J. A., Brunet-Galmés, I., Lalucat, J., Nogales, B. & Bosch, R. MiniUIB, a novel minitransposon-based system for stable insertion of foreign DNA into the genomes of Gram-negative and Gram-positive bacteria. *Appl. Environ. Microbiol.* **79**, 1629–1638 (2013).
10. Tropea, J. E., Cherry, S. & Waugh, D. S. Expression and purification of soluble His_6 -tagged TEV protease. in *High Throughput Protein Expression and Purification, Methods and Protocols* (ed. Doyle, S. A.), Humana Press, 297–307 (2009).
11. Wilson, W. H., Carr, N. G. & Mann, N. H. The effect of phosphate status on the kinetics of cyanophage infection in the oceanic cyanobacterium *Synechococcus* sp. WH7803. *J. Phycol.* **32**, 506–516 (1996).
12. Ksibe, A. Z. Zinc on the move: Insights towards understanding zinc homeostasis in the open ocean cyanobacterium *Synechococcus* sp. WH8102 (University of Warwick, Coventry, 2016).
13. Barnett, J. P. *et al.* Mining genomes of marine cyanobacteria for elements of zinc homeostasis. *Front. Microbiol.* **3**, 142 (2012).
