# Supplementary figures and images for: A single sensor controls large variations in zinc quotas in a marine cyanobacterium
Source: Nat Chem Biol. 2022 Jun 9;18(8):869–77. doi: 10.1038/s41589-022-01051-1 (PMC9337993; doi:10.1038/s41589-022-01051-1)

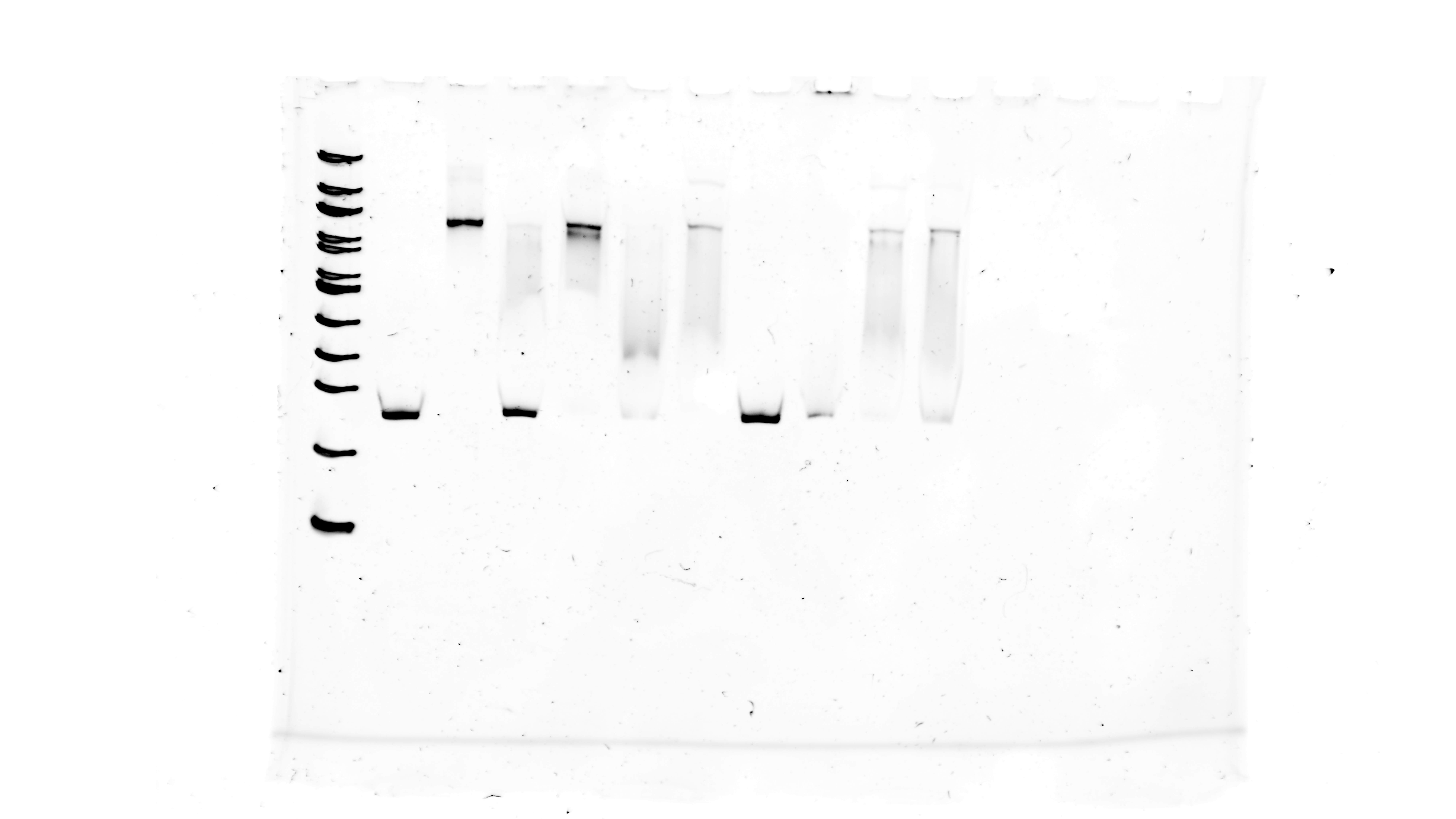

Supplement: Source Data Fig. 1 — Unprocessed gel. [file 41589_2022_1051_MOESM3_ESM.tif]

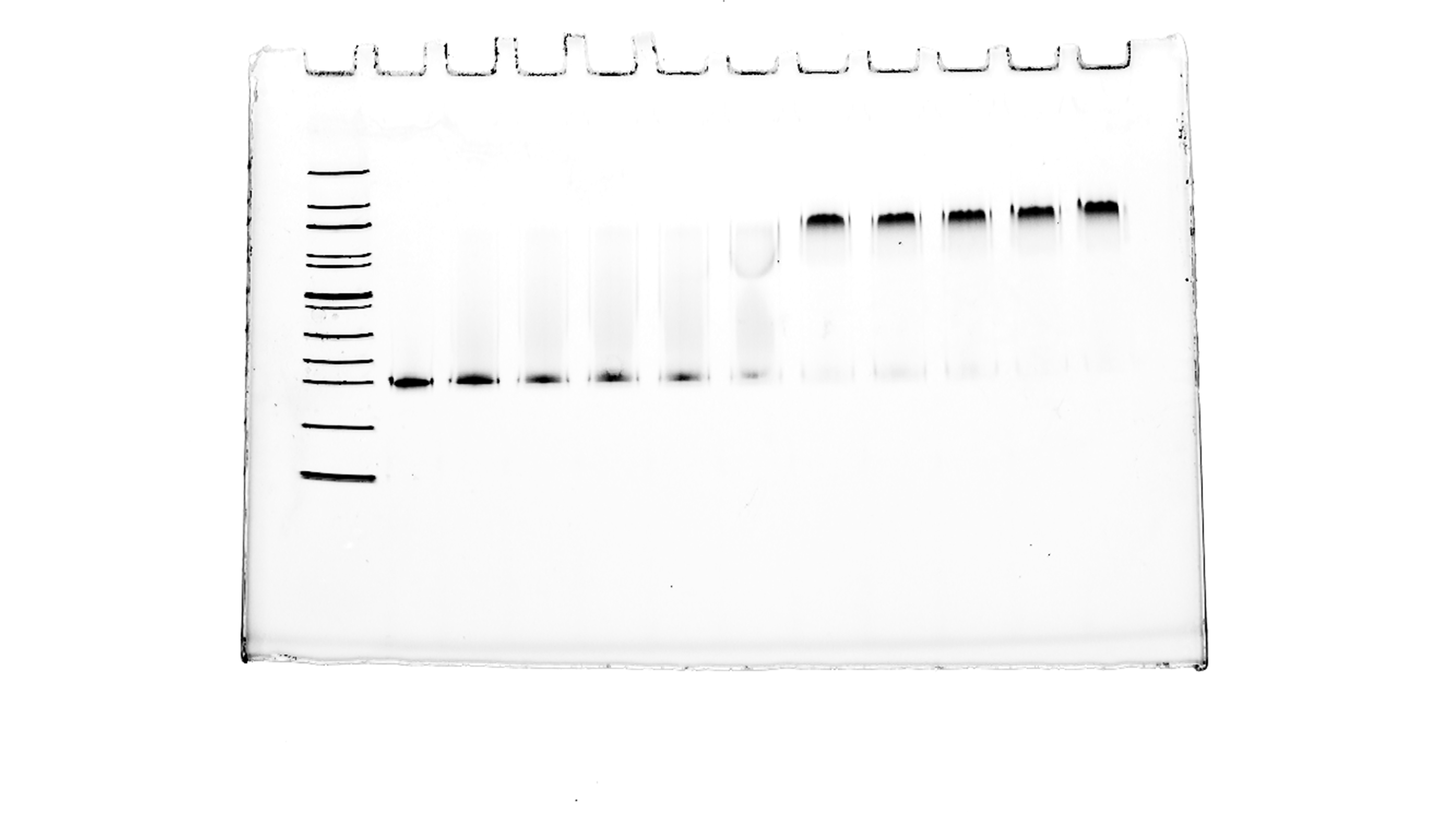

Supplement: Source Data Fig. 4 — Unprocessed gels. [file 41589_2022_1051_MOESM4_ESM.zip › SourceData_Figure_4/SourceData_4f_bottom.tif]

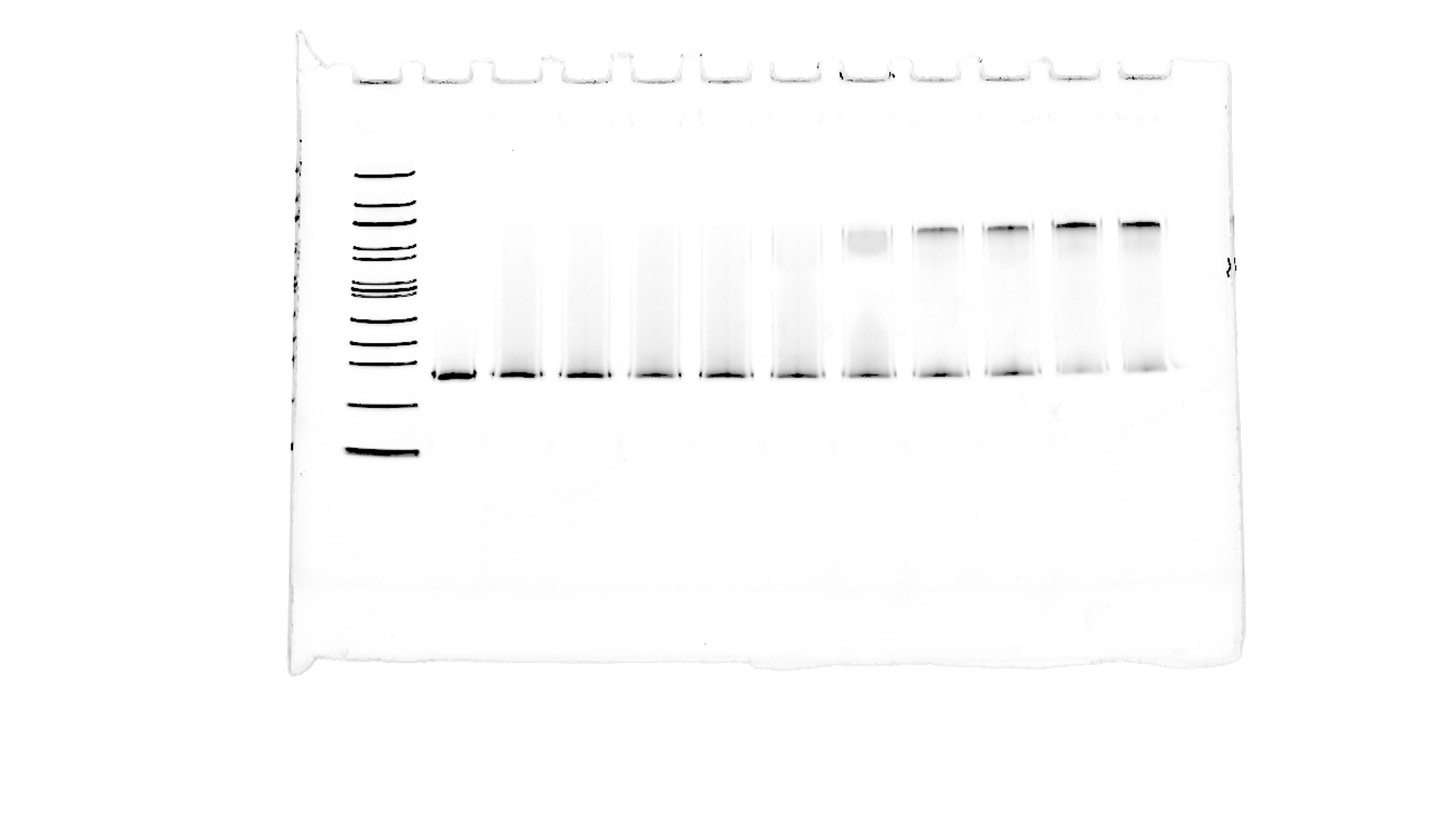

Supplement: Source Data Fig. 4 — Unprocessed gels. [file 41589_2022_1051_MOESM4_ESM.zip › SourceData_Figure_4/SourceData_4f_top.tif]

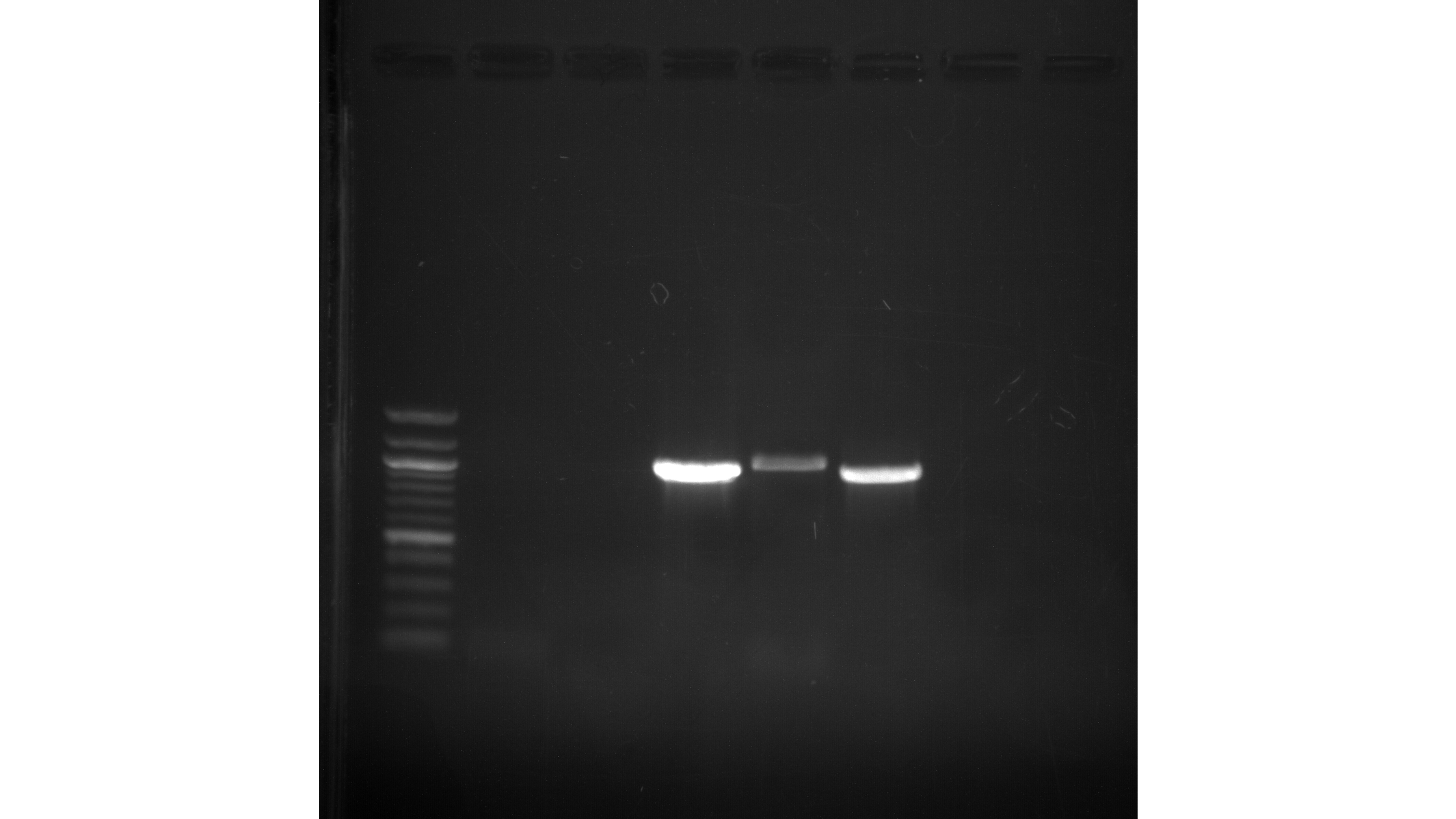

Supplement: Source Data Extended Data Fig. 1 — Unprocessed gel. [file 41589_2022_1051_MOESM5_ESM.tif]

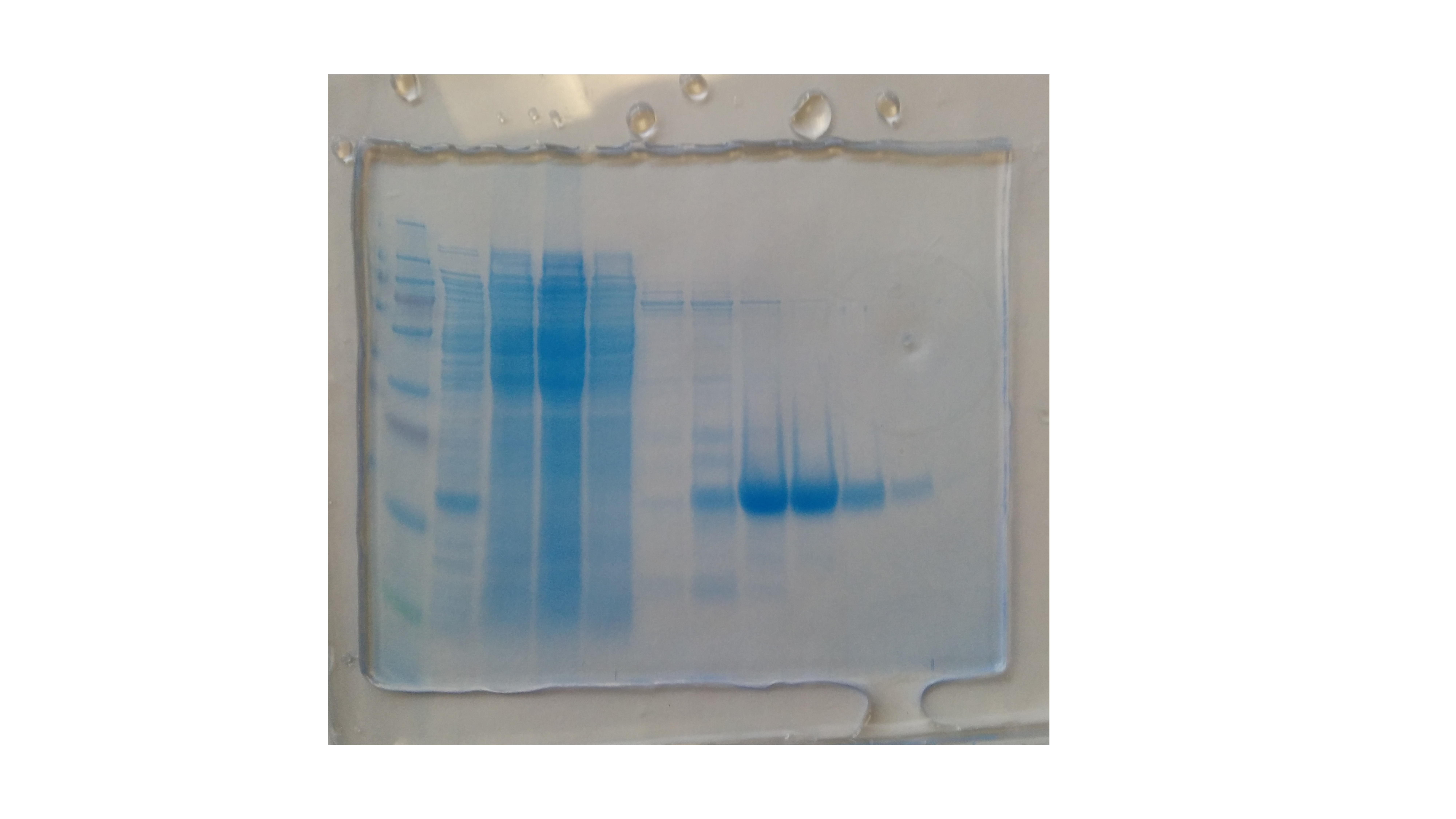

Supplement: Source Data Extended Data Fig. 3 — Unprocessed gels. [file 41589_2022_1051_MOESM6_ESM.zip › SourceData_ED_Figure_3/SourceData_ED3a_1.tif]

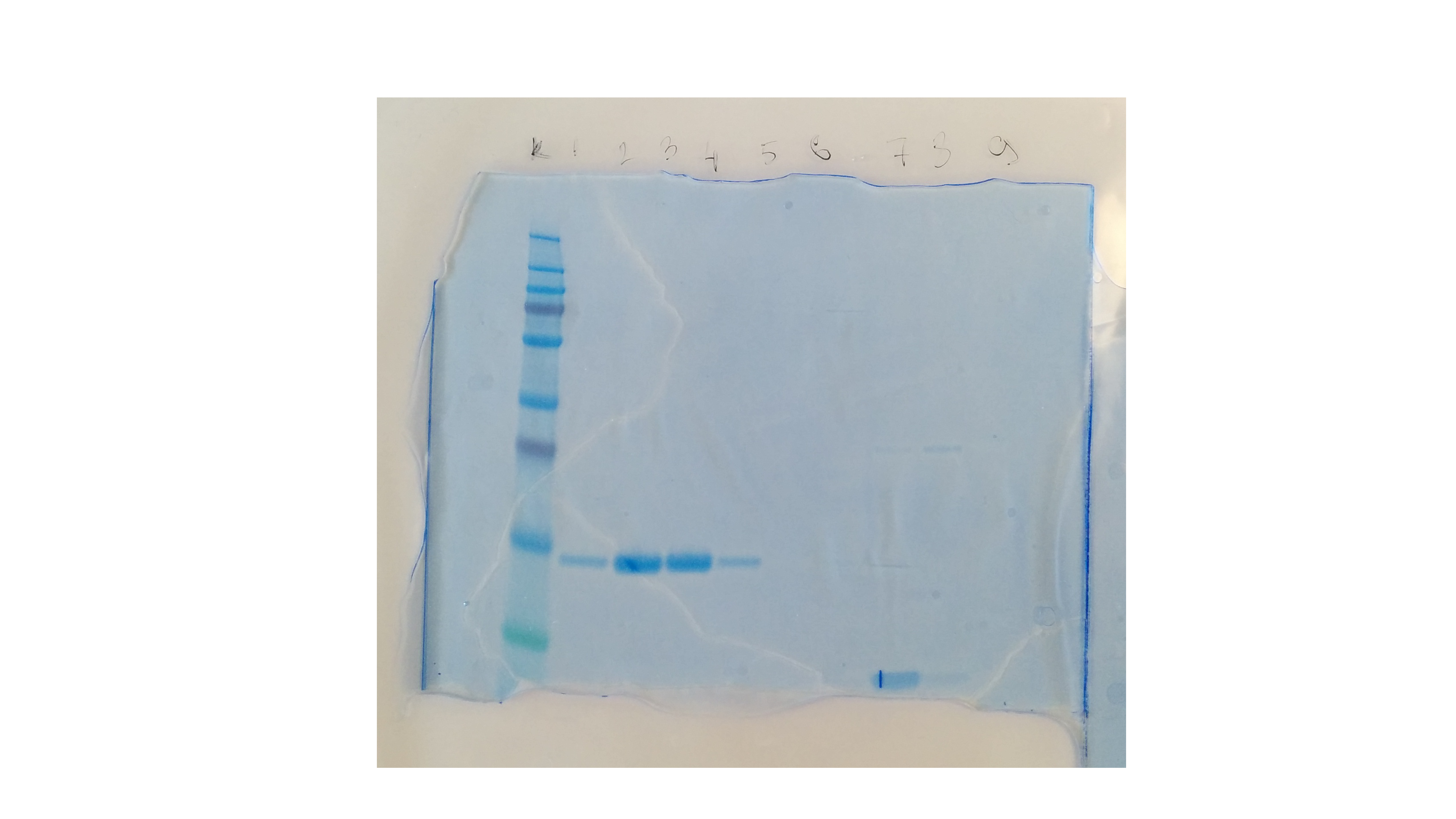

Supplement: Source Data Extended Data Fig. 3 — Unprocessed gels. [file 41589_2022_1051_MOESM6_ESM.zip › SourceData_ED_Figure_3/SourceData_ED3a_2.tif]

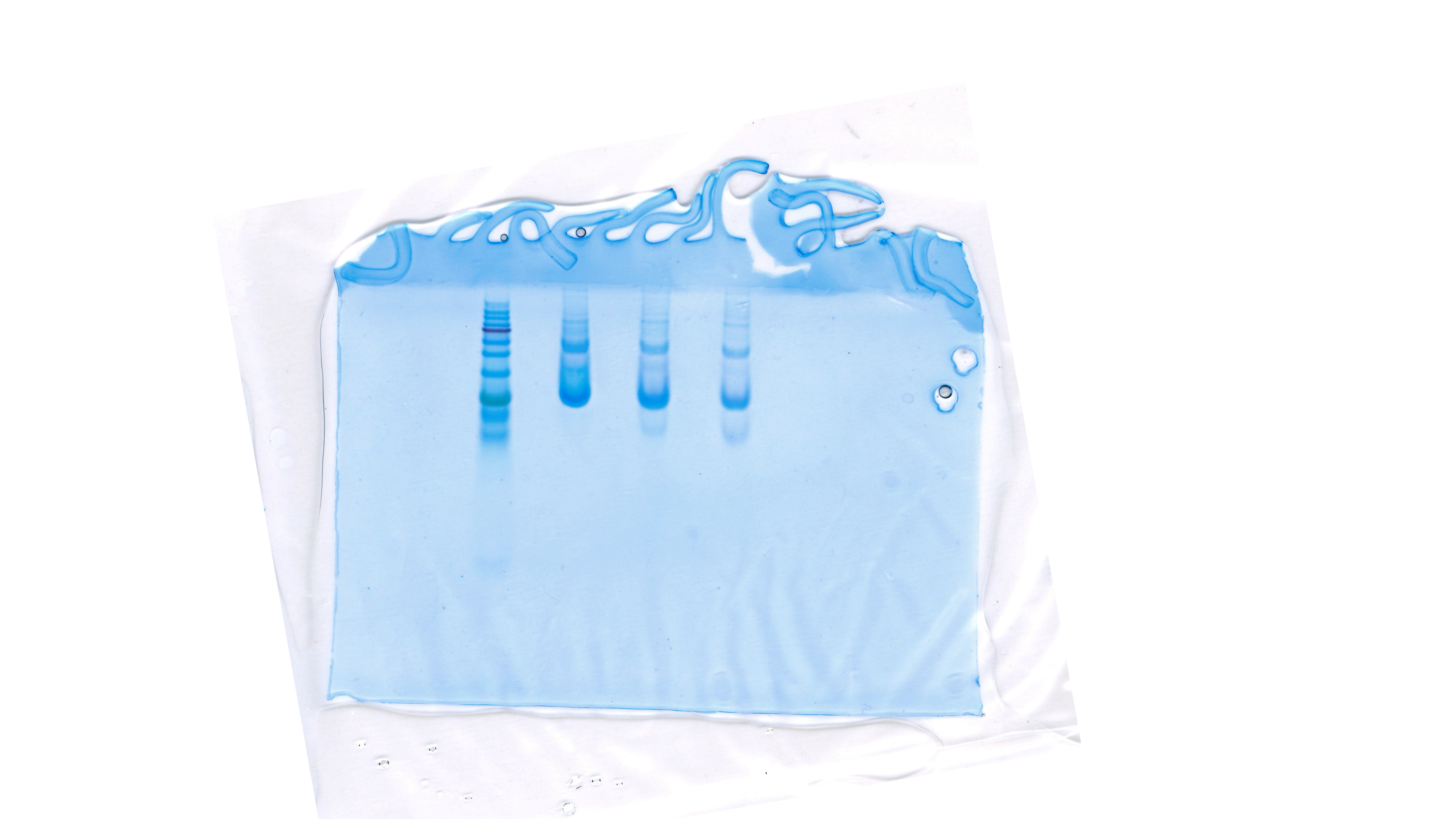

Supplement: Source Data Extended Data Fig. 3 — Unprocessed gels. [file 41589_2022_1051_MOESM6_ESM.zip › SourceData_ED_Figure_3/SourceData_ED3b.tif]

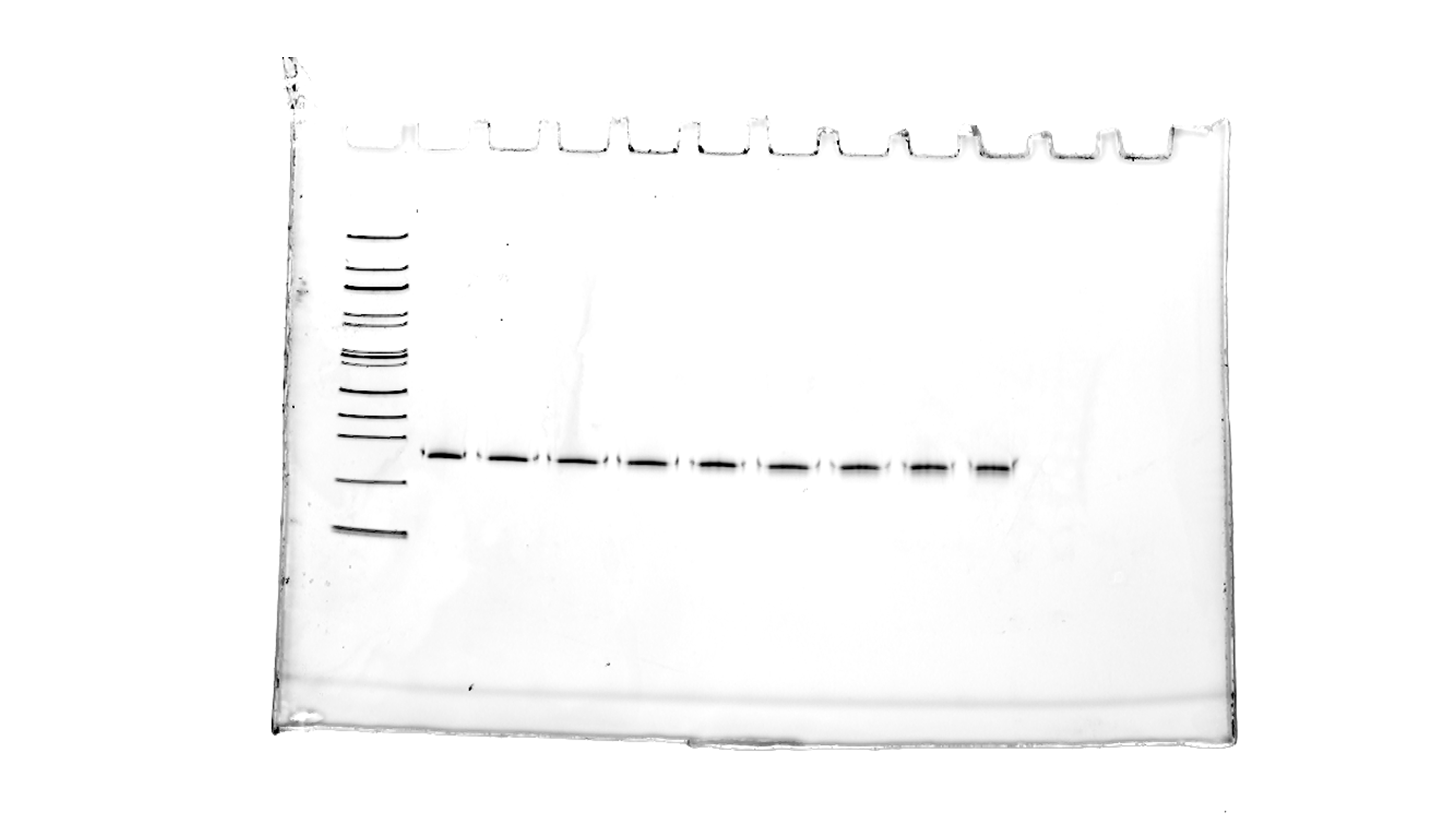

Supplement: Source Data Extended Data Fig. 5 — Unprocessed gel. [file 41589_2022_1051_MOESM7_ESM.tif]
